# A WRKY transcription factor, *SlWRKY75*, positively regulates tomato (*Solanum lycopersicum* L.) resistance to *Ralstonia solanacearum*

**DOI:** 10.3389/fpls.2025.1704937

**Published:** 2025-10-30

**Authors:** Na Chen, Lingzeng Lv, Lian Duan, Jiajun Wu, Qin Shao, Xiaopeng Li, Qineng Lu

**Affiliations:** School of Life Sciences and Environmental Resources, Yichun University, Yichun, China

**Keywords:** tomato, *SlWRKY75*, transcription factor, *Ralstonia solanacearum*, hormone signaling pathway

## Abstract

WRKYs are a unique family of plant-specific transcription factors. Research has proven that WRKY transcription factors play essential roles in regulating plant growth and development as well as biotic and abiotic stress responses. However, the role of WRKY proteins in regulating the resistance of tomato (*Solanum lycopersicum* L.) to bacterial wilt caused by *Ralstonia solanacearum* remains unclear. Our previous study showed that *R. solanacearum* significantly upregulates *SlWRKY75* expression in tomato. In this study, subcellular localization revealed that SlWRKY75 is located in the nucleus, while the transcriptional activation assay indicated that SlWRKY75 acts as a transcriptional activator. To investigate the functional role of SlWRKY75, we generated overexpression transgenic lines. After inoculation with *Ralstonia solanacearum*, these *SlWRKY75*-overexpressing plants displayed significantly enhanced resistance compared to the control plants. This enhanced resistance was supported by several physiological and molecular indicators: the transgenic plants showed better growth, elevated activity of key antioxidant enzymes, increased jasmonic acid (JA) accumulation, and upregulation of genes involved in JA biosynthesis and signaling. The *SlWRKY75*-overexpressing plants also showed decreased levels of hydrogen peroxide (H_2_O_2_), superoxide anion (O_2_^–^), and salicylic acid (SA) and decreased expression of SA synthesis-related and signal response-related genes. Meanwhile, knocking out of *SlWRKY75* via CRISPR/Cas9 system resulted in an opposite trend. Yeast two-hybrid (Y2H) and co-immunoprecipitation (Co-IP) assays confirmed an interaction between SlWRKY75 and SlMYC2, wherein the SlWRKY75 binds to the W-box element in the *SlMYC2* promoter and enhances its expression. These results indicate that the transcription factor *SlWRKY75* positively regulates tomato resistance to bacterial wilt by enhancing the activities of antioxidants and disease resistance enzymes, regulating JA and SA signaling pathways, and modulating reactive oxygen species (ROS) homeostasis. Collectively, our findings not only elucidate a novel SlWRKY75-SlMYC2 regulatory module that fine-tunes hormone signaling and antioxidant defense in tomato immunity, but also highlight the potential of *SlWRKY75* as a valuable gene resource for breeding bacterial wilt-resistant tomato cultivars.

## 1 Introduction

Tomato (*Solanum lycopersicum* L.) is one of the most widely cultivated vegetables worldwide, and it is valued for its high nutritional content, antioxidant properties, and economic value (Meshram and Adhikari, 2024; Rajan et al., 2022). During growth and development, tomato is highly susceptible to bacterial wilt, a typical soil-borne disease caused by *Ralstonia solanacearum*. Bacterial wilt is one of the most destructive plant diseases globally (Chen et al., 2020). *R. solanacearum* can infect over 200 plant species across 50 families, especially those in the Solanaceae family such as tomato, eggplant, and potato (Mansfield et al., 2012; Kim et al., 2016). In tomato, the disease severely affects the quality and causes direct yield losses of up to 20%–50% (Ijaz et al., 2024). Typically, *R. solanacearum* invades the plant xylem through root cell gaps and wounds and rapidly multiplies from the point of invasion. Subsequently, extracellular polysaccharides are produced that facilitate *R. solanacearum* colonization but hinder the plant’s water absorption and transportation abilities. These changes eventually lead to plant dehydration, wilting, and death (Peeters et al., 2013; Chen et al., 2025; Wang et al., 2025). Traditional breeding approaches and chemical control strategies have demonstrated limited success in controlling bacterial wilt, posing serious threats to human health and the environment (Yadav et al., 2022; Gowtham et al., 2024). Notably, genetic engineering through the expression of key disease-resistant genes and developing novel cultivars with enhanced disease resistance is an effective way to manage the disease (Fradin et al., 2009). Understanding the potential physiological and molecular mechanisms associated with tomato defense against *R. solanacearum* infection is crucial to developing more elite resistant cultivars.

Plants have evolved complex defense systems to recognize and resist the invading pathogens (Wang et al., 2018). The defense is achieved primarily by regulating the expression of pathogen response genes through the activities of various transcription factors such as WRKY, bHLH, NAC, AP2/ERF, MYB, and bZIP. These transcription factors also participate in signaling pathways regulated by plant hormones, such as abscisic acid (ABA), salicylic acid (SA), jasmonic acid (JA), and ethylene (ET), and reactive oxygen species (ROS). Plants rely on a complex immune signaling network to defend against pathogen invasion. Among them, the SA and JA signaling pathways are two core pillars (Hou and Tsuda, 2022). These two pathways do not operate independently; there is extensive cross-talk between them, especially the classic antagonistic effect, where the activation of one pathway inhibits the other pathway, providing a strategy for plants to finely regulate the allocation of immune resources (Pieterse et al., 2012; Zhu et al., 2024). In the interaction between plants and *R. solanacearum*, the crucial role of SA has been clearly identified, and studies have found that exogenous application of SA can significantly enhance the resistance of plants to bacterial wilt, manifested by the rapid activation of defense-related genes (such as *PR1*) and the effective control of bacterial populations (Chen et al., 2016; Tang et al., 2017; Zhang et al., 2023a). Conversely, the role of JA is more complex: its antagonism with SA may facilitate susceptibility, but in some cases, JA signals may also exert a positive effect by inducing defense genes (He et al., 2023a; Jiang et al., 2025). MYC2 is the core transcription factor of the JA signaling pathway and is the hub for coordinating the cross-talk between JA and other hormone signals (Luo et al., 2023). Xiao et al. (2024) reported that after knocking out the tobacco gene *NtMYC2a* by CRISPR/Cas9, *NtJAR1* and *NtCOI1* in KO-NtMYC2a plants were downregulated to regulate the JA signaling pathway, resulting in a significant reduction in the resistance of tobacco to *R. solanacearum*. This indicates that *NtMYC2a* plays an important role in regulating tobacco resistance to bacterial wilt. However, the upstream molecular mechanism regulating the expression of *SlMYC2* under *R. solanacearum* infection remains poorly understood. In other words, the transcription factors serve as key components in coordinating the multifaceted responses of plants to environmental stress (Liu et al., 2024; Ottaviani et al., 2025). WRKY is one of the most prominent families of plant transcription factors, and members of the WRKY family typically contain one or two WRKY domains at the C-terminus (each with a length of 60 amino acids) and a conserved amino acid sequence WRKYGQK at their N-terminus (Eulgem et al., 2000; Fan et al., 2025). The WRKY domain contains a zinc finger motif, which can be either C_2_H_2_ type (CX_4-5_CX_22-23_HXH) or C_2_HC type (CX_7_CX_23_HXC) (Eulgem et al., 2000; Rushton et al., 2010). In plants, WRKY proteins activate or inhibit the expression of target genes by recognizing and binding to the W-box (C/T)TGAC(C/T) sequence in their promoter (Dong et al., 2024). Based on the number of WRKY domains and the type of zinc finger motif, WRKY proteins are classified into three phylogenetically distinct groups: group I contains two WRKY domains and a CX_4-5_CX_22-23_HXH zinc finger motif; group II contains one WRKY domain and a CX_4-5_CX_22-23_HXH zinc finger motif; and group III contains one WRKY domain and a CX_7_CX_23_HXC zinc finger motif. The phylogenetic analysis has further classified group II of WRKY proteins can be further divided into five subgroups: IIa, IIb, IIc, IId, and IIe (Rushton et al., 2010; Song et al., 2023).

WRKY transcription factors are well-known regulators of diverse plant processes, including development, and responses to both biotic and abiotic stresses (Fang et al., 2024; Zhang et al., 2024; Su et al., 2025; Zhou et al., 2025). In recent years, several researchers have investigated the role of WRKY transcription factors in regulating plant disease resistance. For example, in strawberry, the content of JA significantly decreases after ripening, while the expression of *FaWRKY25* increases, leading to an increase in its susceptibility to *Botrytis cinerea*. Further study indicates that *FaWRKY25* plays a crucial role in regulating strawberry’s resistance to *B. cinerea* and may negatively regulates the JA-mediated resistance pathway (Jia et al., 2020). Jiang et al. (2021) reported that in tobacco (*Nicotiana benthamiana*), *NbWRKY40* was significantly downregulated after tomato mosaic virus (ToMV) infection. Further research confirmed that *NbWRKY40* regulates the plant’s resistance to ToMV by regulating the level of SA, resulting in callose deposition at the neck of intercellular connections and thereby inhibiting the movement of the virus. In pepper, *R. solanacearum* and exogenous methyl jasmonate (MeJA) are known to significantly induce *CaWRKY22b.* Besides, the overexpression of *CaWRKY22b* led to intense allergic response-like cell death, accumulation of hydrogen peroxide (H_2_O_2_), and upregulation of defense and JA-responsive genes (*CaHIR1*, *CaPO2*, *CaBPR1*, and *CaDEF1*). Conversely, *CaWRKY22b* silencing reduced the resistance of peppers to *R. solanacearum* and upregulated the expression of defense and JA-responsive genes. Further studies have confirmed that CaWRKY22b activates *CaDEF1* expression by directly binding to its promoter. These observations confirmed the role of *CaWRKY22b* in positively regulating pepper’s resistance to *R. solanacearum* by regulating the expression of the JA-responsive gene *CaDEF1* (Shi et al., 2024). Additionally, using the virus-induced gene silencing (VIGS) approach, Zhang et al. (2025a) demonstrated that *SmWRKY30* positively regulates the resistance response of eggplant to bacterial wilt. These studies proved that WRKY transcription factors are widely involved in the plant disease resistance response process.

The involvement of WRKY transcription factors in responding to both biotic and abiotic stresses is well-documented in tomato. For example, Chen et al. (2024) found that overexpression of *SlWRKY6*, a member of the WRKYII-b group, enhanced drought tolerance by strengthening antioxidant defense and stomatal closure via ABA signaling in tomato. Wang et al. (2024) found that a group II WRKY transcription factor, *SlWRKY50*, enhances cold resistance of tomato by regulating the biosynthesis of JA. Moreover, they found that the JA signal regulates the expression of *SlWRKY50* through the transcriptional activation of *SlMYC2*. Similarly, Liu et al. (2025) reported that the SlWRKY42-SlMYC2 module synergistically enhances the saline-alkali tolerance of tomato by activating JA signaling and the spermidine biosynthetic pathway. Kumar et al. (2023) reported that SlWRKY16 and SlWRKY31 act as negative regulators of tomato defense after root-knot nematode (*Meloidogyne javanica*) infection. Shu et al. (2021) found that the overexpression of *SlWRKY46* in tomato increased susceptibility to *B. cinerea*. Detailed analysis revealed that *SlWRKY46* probably plays a negative regulatory role during the infection of *B. cinerea* by inhibiting the activities of antioxidants and disease-resistant enzymes, regulating the SA and JA signaling pathways, and modulating ROS homeostasis. Wang et al. (2023) reported that *ShWRKY81*, from wild tomato *Solanum habrochaites* LA1777, plays a positive role in tomato powdery mildew resistance. Zhao et al. (2025) confirmed that SlWRKY71 can activate the expression of *SlDCD1*, promotes the production of endogenous hydrogen sulfide (H_2_S), and thereby enhances tomato’s resistance to *Pseudomonas syringae* pv. *tomato* DC3000 (*Pst*DC3000). Although WRKY transcription factors were comprehensively identified in tomato, only a few WRKY genes have been identified to respond to *R. solanacearum* infection (Cui, 2018; Dang et al., 2023; Luo, 2024; Shui et al., 2024). Besides, the molecular mechanism associated with tomato’s resistance to *R. solanacearum* infection remains unclear.

Previous study showed that tomato WRKY75 play an important role in response to abiotic stresses (drought or heat) and biotic stresses (Colorado potato beetle larvae infestation, *Pseudomonas syringae* or *B. cinerea* infection) (López-Galiano et al., 2018; Yang et al., 2024a; Zhang et al., 2025b). In addition, our previous study showed that *SlWRKY75* cloned from tomato BY 1–2 plants exhibits differential expression under *R. solanacearum* infection and MeJA or SA treatment. Besides, VIGS demonstrated that silencing *SlWRKY75* reduced tomato resistance, suggesting that it functions as a positive regulator in the defense against *R. solanacearum* (Chen et al., 2023). However, the specific molecular mechanisms underlying this regulation remain unknown. In particular, it is unclear whether SlWRKY75 directly modulates defense-related hormone signaling pathways (e.g., JA and SA) and what its direct downstream target gene are. Given its pronounced transcriptional induction by both the pathogen and defense hormones, SlWRKY75 represents a highly promising candidate for deciphering the transcriptional immune network against bacterial wilt. Therefore, we hypothesized that SlWRKY75 acts as a positive regulator by interacting with the JA signaling pathway, specifically through the key transcription factor *SlMYC2*, to orchestrate the defense response against *R. solanacearum*. To test this hypothesis, the present study combined genetic, biochemical, and molecular approaches to: 1) confirm the positive role of *SlWRKY75* in resistance using overexpression and konckout lines; 2) determine how *SlWRKY75* modulates JA/SA hormone signaling and ROS homeostasis; and 3) identify if SlWRKY75 directly interacts with SlMYC2 and binds to the *SlMYC2* promoter to activate its expression.

## 2 Materials and methods

### 2.1 Plant materials, growth conditions, and pathogen inoculation assays

Two *S. lycopersicum* inbred lines, Hm 2–2 (resistant to *R. solanacearum*) and BY 1–2 (susceptible to *R. solanacearum*), described by Chen et al. (2022), were used as the control plants in this study. The *SlWRKY75* transgenic plants and the *slwrky75* mutants were generated in BY 1–2 and Hm 2–2 backgrounds, respectively. All plants were grown in a commercial potting mix (peat:perlite = 3:1) under controlled conditions in a growth chamber at 28-30°C for 14 h (light) and 18-20°C for 10 h (dark). The relative humidity was maintained at 60-70%. Plants were watered every two days to maintain soil moisture without waterlogging.

At the seven–eight leaf stage, healthy tomato seedlings with consistent growth were selected for inoculation. The root systems were carefully wounded to facilitate bacterial entry. Specifically, five superficial wounds (approximately 2-3 mm in depth) were made on the main roots of each plant using a sterile scalpel. The wounded roots were then immersed in a suspension of *R. solanacearum* strain [1×10^8^ colony forming units (cfu)/mL] for 30 min (Chen et al., 2022). After inoculation, the leaf samples were collected at 0, 48 h for phenotyping, morphological and physiological analyses, and RNA isolation. Three independent experiments were carried out.

### 2.2 Subcellular localization assay

The subcellular localization was analyzed according to the method reported by Bi et al. (2023). The full-length coding sequence (CDS) of *SlWRKY75* was cloned into the pCAMBIA1300 vector to generate the 35S:SlWRKY75-GFP (green fluorescent protein) construct for the localization analysis. The predicted molecular weight of the SlWRKY75 protein is approximately 19.9 kDa, and the resulting SlWRKY75-GFP fusion protein is approximately 46.8 kDa (19.9 kDa + 26.9 kDa). The absence of a canonical N-terminal signal peptide in SlWRKY75 was confirmed using the SignalP software (version 6.0), suggesting its localization is not guided by the classical secretory pathway. The recombinant vector was transformed into the *Agrobacterium tumefaciens* strain GV3101. Then, the Agrobacterium cells carrying the 35S:SlWRKY75-GFP construct or the 35S:GFP empty vector were infiltrated into the leaves of four-week-old *N. benthamiana* plants. After 48 h of incubation, the GFP fluorescence in the injection area of the leaves was observed using a laser confocal microscope (C2-ER, Nikon, Japan). For each construct, at least three independent plants were infiltrated, and fluorescence was examined in multiple epidermal cells from a minimum of three different leaf spots per plant. Each experiment was repeated three times with consistent results. The primers used to construct the plasmids are listed in **Supplementary Table 1**.

### 2.3 Transcriptional activation assay

The transcriptional activity of SlWRKY75 was evaluated using a yeast detection system (Peng et al., 2025). The CDS of *SlWRKY75* was cloned into the pGBKT7 vector at the *Eco*RI and *Bam*HI restriction sites. The recombinant plasmid pGBKT7-SlWRKY75 was transformed into the yeast strain AH109 (Weidi, Guangzhou, China). The transformed yeast cells were cultured on synthetical defined (SD) medium without tryptophan (SD/-Trp) or SD medium without histidine, tryptophan, and adenine (SD/-His/-Trp/-Ade) at 30°C for 3 days, and the α-galactosidase activity was determined using X-α-galactose (X-α-gal) as the substrate. In this assay, the combination of pGADT7-T and pGBKT7-53 was used as the positive control, while pGBKT7-empty was used as the negative control. All experiments were repeated three times. The primers used to construct the plasmids are listed in **Supplementary Table 1**.

### 2.4 Construction of the overexpression and mutant lines

To generate the *SlWRKY75*‐overexpressing tomato plants, the CDS of *SlWRKY75* was cloned into the *Bam*HI and *Sac*I restriction sites of the pBI121 expression vector with the 35S promoter and three flag tags. The sequence-verified recombinant construct was used to genetically engineer tomato BY 1–2 through *Agrobacterium*‐mediated transformation (Liu et al., 2025). Primary transgenic plants (T_0_ generation) were initially screened by polymerase chain reaction (PCR) using primers specific to the vector backbone to confirm the presence of the T-DNA. To quantify the overexpression level of *SlWRKY75*, quantitative real-time PCR (qRT-PCR) was performed. Based on the qRT-PCR analysis, several independent T_0_ lines showing high levels of *SlWRKY75* transcript accumulation were selected and self-pollinated to produce the T_1_ generation. In the T_1_ generation, three independent homozygous lines (OE–2, OE–4, and OE–7) with higher *SlWRKY75* expression were finally used for further studies.

Similarly, to generate the *slwrky75* mutants, two small guide RNAs were designed based on the target site in the *SlWRKY75* sequence using the online website CRISPR-GE (http://skl.scau.edu.cn/help/). PCR was performed to obtain a DNA fragment containing the gRNA-scaffold and the target site sequence using the pCBC-DT1T2 vector (gRNA expression cassette) as the template (Xing et al., 2014). Then the DNA fragment was cloned into the *Bsa*I site of the pHSN401 vector. The obtained plasmid was verified by the DNA sequencing, introduced into *A. tumefaciens* strain EHA105, and used to transform the Hm 2–2 plants (Yan et al., 2020). To screen for mutant plants, genomic DNA was extracted from T_0_ regenerated plants. The target region was amplified by PCR and subjected to Sanger sequencing. The sequencing chromatograms were analyzed using the DSDecode online tool to decipher the exact mutation types (insertions or deletions). Homozygous or biallelic mutants were identified by the presence of a single, clean peak in the sequencing chromatogram at the target site, indicating identical edits on both alleles. Potential off-target sites for each sgRNA were predicted using the Cas-OFFinder online tool. The top 3 potential off-target sites with the highest similarity scores were selected for analysis. PCR amplification and sequencing of these loci were performed on the final selected homozygous mutant lines to check for any unintended mutations. DNA sequencing and qRT-PCR were performed to identify and confirm the mutations. Finally, three mutant lines (cr–5, cr–8, and cr–35) of the T_1_ generation were used for further studies. The primers used to construct the plasmids are listed in **Supplementary Table 1**.

### 2.5 RNA extraction and qRT-PCR analysis

Total RNA was extracted from tomato plants using the HiPure Total RNA Mini Kit (Magen, Guangzhou, China). Then 1 μg of total RNA was used as the template for complementary DNA (cDNA) synthesis with the HiScript II 1st Stand cDNA Synthesis Kit (+gDNA wiper) (Vazyme, Nanjing, China). The obtained cDNA was diluted 5-fold and used to perform qRT-PCR on the StepOne Real-Time PCR (Applied Biosystems, Thermo Fisher, USA). The PCR system was established using the 2×RealStar Fast SYBR qPCR Mix (High ROX) Kit (GenStar, Beijing, China), following the manufacturer’s instructions. Then qRT-PCR was carried out using the following program: 95°C for 2 min; 95°C for 15 s, 60°C for 20 s (40 cycles); melting curve (automatically set by the instrument). Three biological replicates and three technical replicates were conducted in this study. The relative expression levels of the tested genes were calculated following the 2^-ΔΔCt^ method (Livak and Schmittgen, 2001), using tomato *SlACTIN* as the internal reference gene to normalize the relative expression levels. The sequences of the primers used for qRT-PCR are given in **Supplementary Table 2**.

### 2.6 Determination of morphological and physiological indicators

After inoculation with *R. solanacearum* and incubation for 48 h, the leaf samples of the control plants (BY 1–2 and Hm 2–2 plants), *SlWRKY75*-overexpressing plants, and the *slwrky75* mutants were collected. For histochemical staining: The accumulation of H_2_O_2_ and O_2_^−^ was visualized by nitrotetrazolium blue chloride (NBT) and 3,3’-diaminobenzidine (DAB) staining, respectively, using commercial kits (Servicebio, Wuhan, China). Briefly, leaf discs were vacuum-infiltrated with the NBT or DAB solution and incubated in the dark. After destaining, the formation of dark blue (NBT) or Brown (DAB) precipitates indicated the presence of H_2_O_2_ or O_2_^−^, respectively. For biochemical assays: The activities of key antioxidant enzymes—including total superoxide dismutase (SOD), peroxidase (POD), catalase (CAT), phenylalanine ammonia-lyase (PAL), and polyphenol oxidase (PPO)—as well as the content of malondialdehyde (MDA) were quantified using commercial assay kits (Jiancheng Bioengineering Institute, Nanjing, China). The assays were performed according to the manufacturers’ protocols, which are based on established spectrophotometric principles: SOD activity was measured by its ability to inhibit the photochemical reduction of nitroblue tetrazolium. POD and CAT activities were determined by monitoring the decomposition of H_2_O_2_ at 470 nm and 240 nm, respectively. PAL activity was assayed by measuring the production of *trans*-cinnamic acid from L-phenylalanine at 290 nm. PPO activity was determined by the increase in absorbance at 420 nm due to the oxidation of catechol. MDA content, an indicator of lipid peroxidation, was measured by its reaction with thiobarbituric acid (TBA) at 532 nm. Additionally, the quantitative contents of H_2_O_2_ and superoxide anion (O_2_^−^) were determined using kits from Solarbio (Beijing, China). H_2_O_2_ and O_2_^−^ contents were quantified using specific chromogenic reactions at 415 nm and 530 nm, respectively. All procedures were conducted in accordance with the manufacturers’ protocols.

### 2.7 Determination of endogenous hormone content

Approximately 0.5 g of leaf sample was collected from tomato plants both before and 48 h after inoculation with *R. solanacearum* and rapidly frozen in liquid nitrogen. The sample was mixed with steel beads, 1 mL of isopropanol-water solution (80:20, V/V, containing an isotope-labeled internal standard mixture), and vortexed for 30 s. The mixture was further homogenized at 40 Hz for 4 min and ultrasonicated in an ice water bath for 5 min. The homogenization and ultrasonication steps were repeated three times. The mixture was stored at -20°C for 1 h and centrifuged at 4°C and 14,000 rpm for 15 min. Then, 800 μL of the supernatant was taken, dried by centrifugation, and re-dissolved in 160 μL of methanol-water solution (50:50, V/V). Again, the sample was centrifuged at 14,000 r/min for 10 min, and the supernatant was collected, passed through a 0.22 μm filter membrane, and analyzed on a Waters ACQUITY I-Class (Waters, Shanghai, China) ultra-high performance liquid chromatograph. The sample was passed through an ACQUITY UPLC HSS T3 (100 × 2.1 mm, 1.8 μm, Waters) column at a temperature of 40°C. Then, mobile phases A and B were used. Mobile phases A consisted of a 0.1% formic acid aqueous solution, and mobile phases B consisted of acetonitrile containing 0.1% formic acid. The injection volume was set at 5 μL. The mass spectrometry parameters were established as follows: curtain gas = 35 psi, ion spray voltage at +5500 V/-4500V, temperature at 550°C, ion source gas 1 at 50 psi, and ion source gas 2 at 55 psi.

### 2.8 Yeast two-hybrid assay

The CDS of *SlWRKY75* was cloned into the pGADT7 vector at the *Eco*RI and *Bam*HI restriction sites. The full‐length CDS of *SlMYC2*, *SlCOI1*, *SlJAZ*, *SlLOXD*, *SlAOS*, *SlAOC*, *SlNPR1*, *SlTGA*, *SlPR1*, *SlPAL*, and *SlICS1* were inserted into the pGBKT7 vector, respectively. The pGADT7-SlWRKY75 and pGBKT7-SlMYC2 (SlLOXD, SlAOS, SlAOC, SlCOI1, SlJAZ, SlNPR1, SlTGA, SlPR1, SlPAL, or SlICS1) recombinant plasmids were co-transformed into the yeast strain AH109 (Weidi, Guangzhou, China). The transformed yeast cells were cultured on SD medium without leucine and tryptophan (SD/-Leu/-Trp) to select for successfully co-transformed yeast colonies. To suppress the self-activation background of SlWRKY75, the competitive inhibitor 3-amino-1,2,4-triazole (3-AT) was added to the selection medium at a concentration of 10 mM, which was determined by titration to be the minimal concentration required to completely inhibit autoactivation while allowing true positives to grow. Therefore, the screening for protein-protein interactions was performed on SD medium without leucine, tryptophan, adenine and histidine (SD/-Leu/-Trp/-Ade/-His) supplemented with 10 mM 3-AT. The plates were incubated at 30 °C for 3 days, and the α-galactosidase activity was determined using X-α-gal as the substrate. In this assay, the following plasmid pairs were used as controls: pGADT7-T with pGBKT7-53 (positive control), and pGADT7-T with pGBKT7-Lam (negative control). All experiments were repeated three times. The primers used to construct the plasmids are listed in **Supplementary Table 1**.

### 2.9 Co-immunoprecipitation assay

The full-length CDS of *SlMYC2* was cloned into the pCAMBIA1300-35S-GFP vector to generate the SlMYC2-GFP construct. The CDS of *SlWRKY75* was fused to the pCAMBIA1300-35S-4MYC vector to produce the SlWRKY75-MYC recombinant construct. Then, the sequence-verified recombinant plasmids were transformed into *A. tumefaciens* GV3101. Subsequently, the cells carrying the vector were co‐injected into the *N. benthamiana* leaves, and these plants were maintained in the dark for 48 h. Total proteins were extracted from the tobacco leaves and incubated with the anti-GFP polymer gel at 4°C for 16 h. Then, the immune complexes were collected by centrifugation and resuspended in 1×SDS-PAGE buffer. Finally, immunoprecipitation of the GFP-fused SlMYC2 was performed using an anti-GFP mouse antibody (Roche), and the proteins were detected using an anti-MYC rabbit antibody (Abclonal) (Chen et al., 2018). The primers used to generate these constructs are listed in **Supplementary Table 1**.

### 2.10 Yeast one-hybrid assay

Reverse transcription PCR (RT-PCR) was performed using specific primers (SlMYC2 pro, see **Supplementary Table S1**) to obtain the promoter sequence of *SlMYC2* (2000 bp upstream of the translation initiation codon of *SlMYC2*). Further, based on the characteristics of the promoter sequence, fragments (SlMYC2 pro: -1 bp to -2016 bp; W1: -1417 bp to -2016 bp; W2: -717 bp to -1367 bp; W3: -517 bp to -716 bp; W4: -7 bp to -516 bp) containing potential W-box *cis*-acting elements at *Sac*I and *Sal*I restriction sites were chemically synthesized and integrated into the pAbAi vector to generate AbAi-SlMYC2 pro, AbAi-pSlMYC2-W1, AbAi-pSlMYC2-W2, AbAi-pSlMYC2-W3, and AbAi-pSlMYC2-W4 reporter vectors, respectively. Similarly, the full-length CDS of *SlWRKY75* was cloned into the pGADT7 vector to obtain the pGADT7-SlWRKY75 effector vector. The vectors containing the pAbAi-SlMYC2 pro (AbAi-pSlMYC2-W1, AbAi-pSlMYC2-W2, AbAi-pSlMYC2-W3, or AbAi-pSlMYC2-W4) construct and the pGADT7-SlWRKY75 construct were co-transformed into the yeast strain Y1H (Weidi, Guangzhou, China). Here the pGADT7-53 and p53-AbAi plasmids were co-transformed into yeast cells and used as a positive control. The empty pGADT7 vector and the corresponding AbAi-SlMYC2 pro vector were co-transformed into yeast cells and used as a negative control. The transformed yeast cells were cultured on SD medium without uracil and leucine (SD/-Ura/-Leu) (with or without 500 ng/mL Aureobasidin A (AbA)) for 3 days at 30°C. All experiments were repeated three times. The primers used to clone the promoter and construct the vectors are listed in **Supplementary Table 1**.

### 2.11 Dual‐luciferase reporter assay

The CDS of *SlWRKY75* was inserted into the pGreenII 62-SK vector to generate the effector plasmid. Meanwhile, a 2000 bp promoter fragment of *SlMYC2* (described above) was cloned into the pGreenII 0800 LUC vector to generate the reporter plasmid (Cao et al., 2025). Then, the effector and reporter plasmids were transfected into *A. tumefaciens* GV3101, respectively. The bacterial cultures carrying the effector and reporter were mixed in equal volumes and concentrations and co-injected into the fully expanded leaves of four-week-old *N. benthamiana* plants. The plants were cultured in the dark for 12 h and then under a 16-h light/8-h dark cycle for 48 h. The luciferase activities due to Firefly luciferase (LUC) and Renilla luciferase (REN) were finally measured using the dual-luciferase assay kit (Promega, Madison, USA). Each experiment was repeated three times. The primers used in this experiment are listed in **Supplementary Table 1**.

### 2.12 Statistical analysis

Data are presented as the mean ± standard error (SE) of three biological replicates. After confirming that the data met the assumptions of normality and homogeneity of variances for ANOVA, significant differences between groups (*P* < 0.05) were assessed by one-way ANOVA with Tukey’s HSD test, as indicated by different letters. All analyses were conducted with GraphPad Prism 8.0.

### 2.13 Accession numbers

Gene sequences for the genes described in this article can be retrieved at the tomato genome initiative database as follows: *SlWRKY75* (Solyc05g015850), *SlACTIN* (Solyc03g078400), *SlMYC2* (Solyc08g076930), *SlLOXD* (Solyc03g122340), *SlAOS* (Solyc04g079730), *SlAOC* (Solyc02g085730), *SlCOI1* (Solyc05g052620), *SlJAZ* (Solyc12g009220), *SlNPR1* (Solyc07g040690), *SlTGA* (Solyc11g064950), *SlPR1* (Solyc01g106620), *SlPAL* (Solyc09g007900), and *SlICS1* (Solyc06g071030).

## 3 Results

### 3.1 SlWRKY75 is localized in the nucleus and induces transcriptional activity

**Figure 1:**
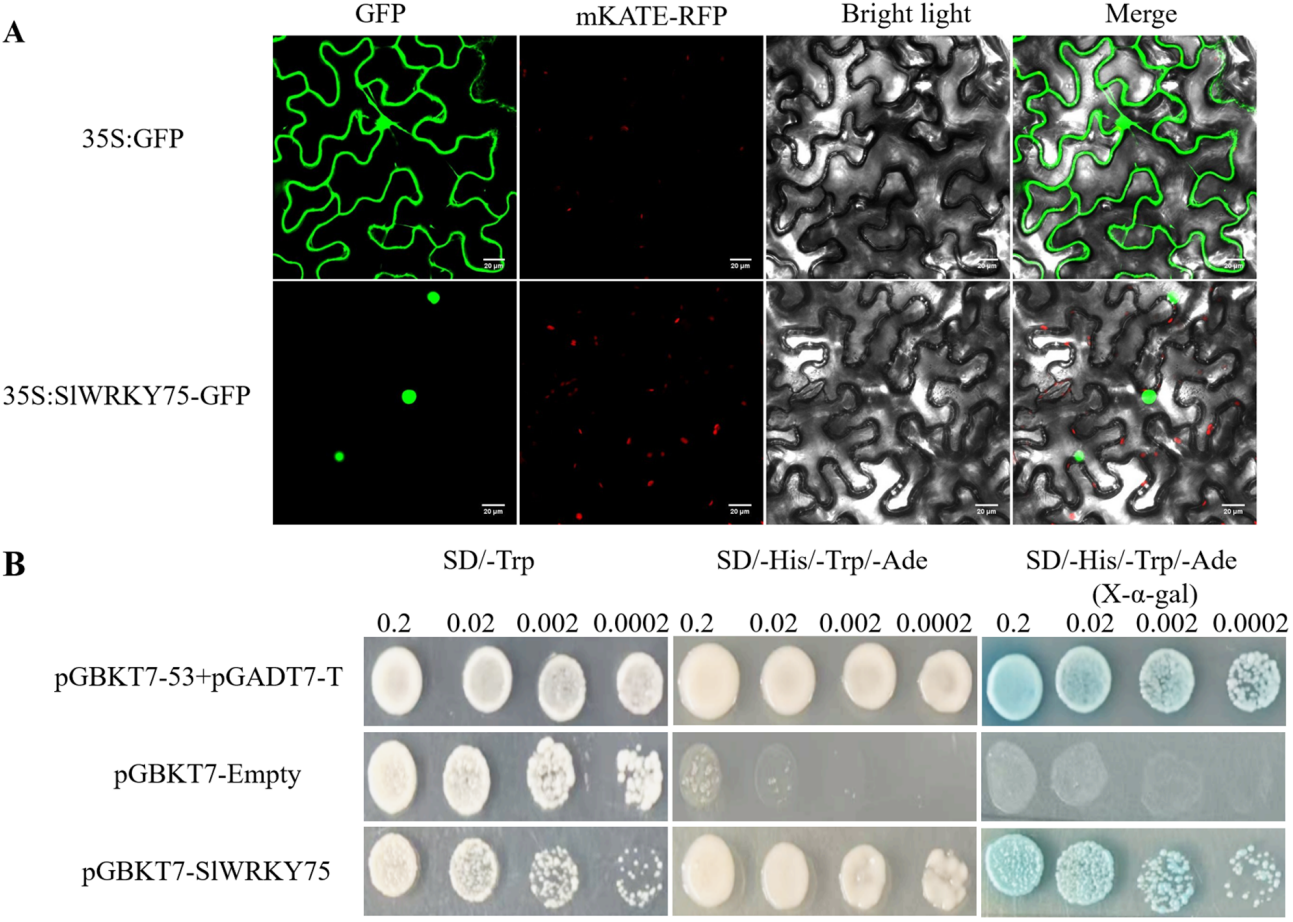
Subcellular localization and transcriptional activation of SlWRKY75. **(A)** Subcellular localization of SlWRKY75 in Tobacco epidermal cells. Scale bar = 20 μm. **(B)** Transcriptional activation of SlWRKY75 in yeast.

The CDS of *SlWRKY75* was cloned into a GFP vector to generate the SlWRKY75::GFP construct and transiently expressed in tobacco (*Nicotiana tabacum*) leaves to determine its subcellular localization. After incubation for 48 h, the GFP signal due to this construct was detected in the nuclear region, while the GFP signal due to the control (35S-GFP) was detected in both the cytoplasm and the nucleus (**Figure 1A**), indicating that SlWRKY75 is a nuclear protein.

Further, the GAL4 yeast expression system was used to detect the transcriptional activation activity of SlWRKY75. The full-length CDS of *SlWRKY75* was introduced into the pGBKT7 vector, and the recombinant vector was transformed into AH109 yeast cells. The transcription activation assay was carried out using the combination of pGADT7-T + pGBKT7-53 as the positive control and pGBKT7-empty as the negative control. All yeast cells grew normally on the SD/-Trp medium. Furthermore, only the cells carrying the combination of pGADT7-T + pGBKT7-53 positive control and those carrying the pGBKT7-SlWRKY75 construct grew and displayed a blue color on the SD/-His/-Trp/-Ade medium or SD/-His/-Trp/-Ade medium containing X-α-gal. Meanwhile, the yeast cells transformed with the pGBKT7-empty vector did not survive on the SD/-His/-Trp/-Ade medium (**Figure 1B**). These observations indicated that SlWRKY75 possesses transcriptional activation activity.

### 3.2 Overexpression of *SlWRKY75* enhanced tomato resistance to *R. solanacearum*

**Figure 2:**
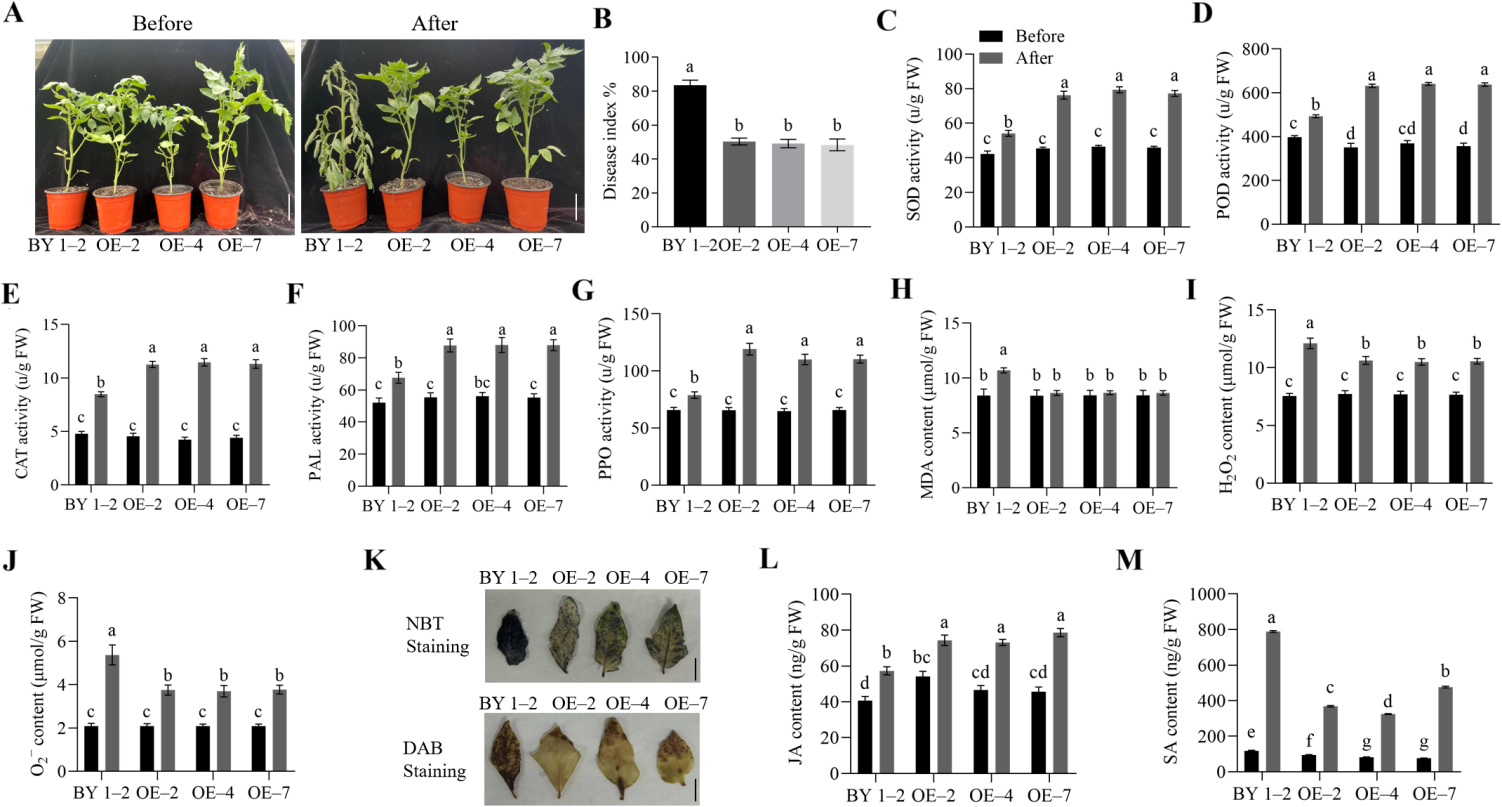
Overexpression of *SlWRKY75* enhanced tomato resistance to *R. solanacearum*. **(A)** Phenotypes of the *SlWRKY75*-overexpressing lines (OE–2, OE–4 and OE–7) and wild-type BY 1–2 plants before and after inoculation with *Ralstonia solanacearum* for 7 d **(B)** The disease index (DI) values of BY 1–2 and *SlWRKY75-*overexpressing lines after inoculation with *R. solanacearum* for 20 d **(C−G)** The activities of superoxide dismutase (SOD), peroxidase (POD), catalase (CAT), phenylalanine ammonia-lyase (PAL), and polyphenol oxidase (PPO) in BY 1–2 and *SlWRKY75*-overexpressing lines before and after inoculation with *R. solanacearum* for 48 h **(H−J)** The contents of malondialdehyde (MDA), hydrogen peroxide (H_2_O_2_), and superoxide anion (O_2_^−^) in BY 1–2 and *SlWRKY75*-overexpressing lines before and after inoculation with *R. solanacearum* for 48 h **(K)** 3,3’‐diaminobenzidine (DAB) and nitrotetrazolium blue chloride (NBT) staining in BY 1–2 and *SlWRKY75*-overexpressing lines after inoculation with *R. solanacearum* for 48 h **(L−M)** The contents of JA and SA in BY 1–2 and *SlWRKY75*-overexpressing lines before and after inoculation with *R. solanacearum* for 48 h Data are means of three biological replicates ± standard error (SE). Different letters indicate statistically significant differences among groups (Tukey’s honest significant difference test, *P* < 0.05). Bars, 4 cm in A and 1 cm in K.

In this study, transgenic tomato plants were generated to assess the role of *SlWRKY75* in regulating the plant defense response to *R. solanacearum*. The CDS of *SlWRKY75* was cloned into the pBI121 vector to generate the pBI121-SlWRKY75 recombinant plasmid (**Supplementary Figure 1A**), and the transgenic plants were obtained in the BY 1–2 background by *Agrobacterium*-mediated transformation. Subsequently, three transgenic lines (OE–2, OE–4, and OE–7) with higher *SlWRKY75* expression were selected based on PCR and qRT-PCR to analyze the pathogen resistance ability (*P* < 0.0001 for OE–2, OE–4, OE–6, and OE–7, *P* = 0.0910 for OE–1, *P* = 0.0151 for OE–3; **Supplementary Figures 1B, C**). The wild-type plant (BY 1–2) and the transgenic lines (OE–2, OE–4, and OE–7) at the seven- to eight-leaf stage were inoculated with the *R. solanacearum* pathogen. One week after inoculation, the transgenic lines showed stronger resistance than the BY 1–2 plants (**Figure 2A**). The disease index (DI) of the three transgenic lines OE–2, OE–4, and OE–7 was 50.41%, 49.19%, and 48.33%, respectively, which were lower than that of the BY 1–2 plants (83.55%; for all comparisons, *P* < 0.0001; **Figure 2B**). This observation indicates that the damage due to *R. solanacearum* infection in the transgenic plants was less severe than that in the BY 1–2 plants. Additionally, compared to their respective untreated controls, 48 h after *R. solanacearum* inoculation, the activities of SOD, POD, CAT, PAL, and PPO in the *SlWRKY75*-overexpressing plants were significantly higher than those in the BY 1–2 plants (*P* < 0.05; **Figures 2C–G**). The contents of MDA, H_2_O_2_, and O_2_^−^ in the leaves of the transgenic lines were significantly lower than those of the BY 1–2 plants (*P* < 0.05; **Figures 2H–J**). Consistently, NBT and DAB staining showed that the transgenic lines had less H_2_O_2_ and O_2_^−^ in their leaves than the BY 1–2 plants (**Figure 2K**). Furthermore, the study showed that the JA levels in both wild-type and transgenic plants significantly increased after inoculation compared to their respective untreated controls. The levels of JA in OE–2, OE–4, and OE–7 were significantly higher than those in the wild-type plants (*P* < 0.05; **Figure 2L**). Similarly, the SA levels in both wild-type and transgenic plants significantly increased after inoculation compared to their respective untreated controls. However, the levels of SA in the OE–2, OE–4, and OE–7 lines were significantly lower than those in the wild-type plants (*P* < 0.05; **Figure 2M**).

### 3.3 Knock out of *SlWRKY75* decreased plants resistance to *R. solanacearum*

**Figure 3:**
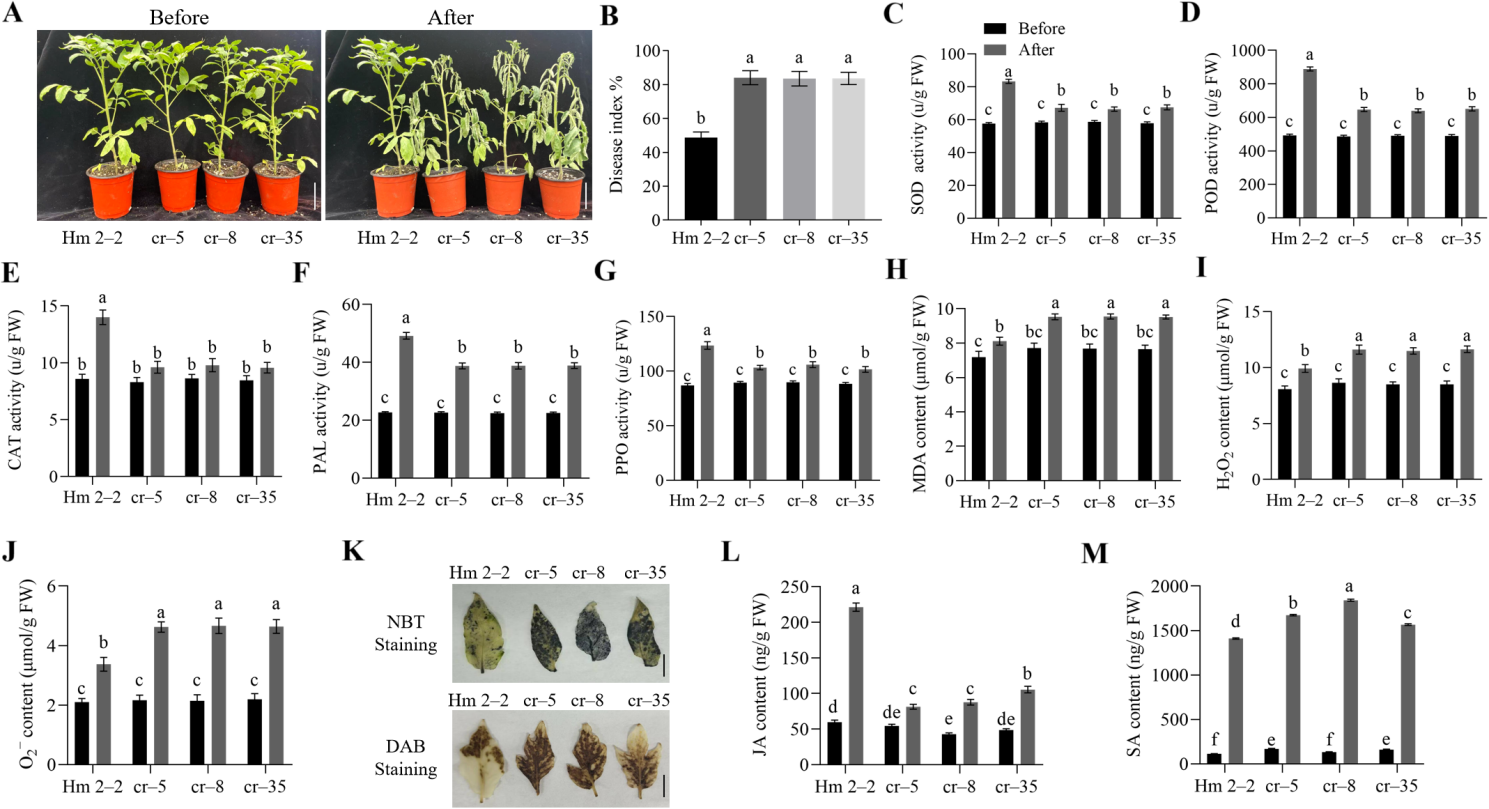
Knock out of *SlWRKY75* decreased tomato resistance to *R. solanacearum*. **(A)** Phenotypes of the *slwrky75* mutant lines (cr–5, cr–8, and cr–35) and wild-type Hm 2–2 plants before and after inoculation with *R. solanacearum* for 7 d **(B)** The disease index (DI) values of Hm 2–2 and *slwrky75* mutants after inoculation with *R. solanacearum* for 20 d **(C−G)** The activities of superoxide dismutase (SOD), peroxidase (POD), catalase (CAT), phenylalanine ammonia-lyase (PAL), and polyphenol oxidase (PPO) in Hm 2–2 and *slwrky75* mutant lines before and after inoculation with *R. solanacearum* for 48 h **(H−J)** The contents of malondialdehyde (MDA), hydrogen peroxide (H_2_O_2_), and superoxide anion (O_2_^−^) in Hm 2–2 and *slwrky75* mutant lines before and after inoculation with *R. solanacearum* for 48 h **(K)** 3,3’‐diaminobenzidine (DAB) and nitrotetrazolium blue chloride (NBT) staining in Hm 2–2 and *slwrky75* mutant lines after inoculation with *R. solanacearum* for 48 h **(L−M)** The contents of jasmonic acid (JA) and salicylic acid (SA) in Hm 2–2 and *slwrky75* mutant lines before and after inoculation with *R. solanacearum* for 48 h Data are means of three biological replicates ± standard error (SE). Different letters indicate statistically significant differences among groups (Tukey’s honest significant difference test, *P* < 0.05). Bars, 4 cm in A and 1 cm in **(K)**.

To further clarify the role of *SlWRKY75* in resisting *R. solanacearum*, we used the CRISPR/Cas9 system and knocked out *SlWRKY75* from the Hm 2–2 tomato variety. Two sites, located in the first exon of *SlWRKY75*, were targeted to develop these mutants (**Supplementary Figure 2A**). A total of 10 independent T_0_ transgenic lines were obtained. Sequencing of the target region revealed that 7 lines carried mutations, yielding a mutation rate of 70%. Using this approach, three types of *slwrky75* homozygous mutants (cr–5: 92 bp deletions; cr–8: 6 bp deletions; cr–35: 4 bp deletions, **Supplementary Figure 2B**) were obtained. Sequencing of the top 3 predicted off-target sites in these homozygous lines confirmed that no off-target mutations were detected. Subsequent qRT-PCR showed that the expression levels of *SlWRKY75* in the *slwrky75* mutants were significantly lower than those in the wild-type Hm 2–2 plants (for all comparisons, *P* < 0.0001; **Supplementary Figure 2C**). Further, the Hm 2–2 plants and the *slwrky75* mutants were inoculated with the *R. solanacearum* to evaluate and confirm the role of this transcription factor in regulating the pathogen-induced damage. One week after inoculation, the mutant plants showed higher susceptibility than the Hm 2–2 plants (**Figure 3A**). The DI values of the cr–5, cr–8, and cr–35 mutant lines were approximately 84.12%, 83.54%, and 83.65%, respectively, while the value of the Hm 2–2 plants was 48.81% (*P* < 0.0001 for all mutant lines compared to Hm 2–2; **Figure 3B**). These observations indicate that the damage due to *R. solanacearum* infection in the mutant plants was more severe than that in the Hm 2–2 plants. Additionally, compared to their respective untreated controls, 48 h after *R. solanacearum* inoculation, the activities of SOD, POD, CAT, PAL, and PPO in the leaves of the mutant plants were significantly lower than those in the Hm 2–2 plants (*P* < 0.05; **Figures 3C–G**). The contents of MDA, H_2_O_2_, and O_2_^−^ in the leaves of the mutant plants were significantly higher than those in the Hm 2–2 plants (*P* < 0.05; **Figures 3H–J**). Consistently, H_2_O_2_ and O_2_^−^ produced in the leaves of the mutant plants were higher than those in the Hm 2–2 plants (**Figure 3K**). Further analysis showed that compared to their respective untreated controls, after inoculation, the JA levels in both wild-type and mutant plants increased; the JA levels in the mutant plants were significantly lower than those in the Hm 2–2 plants (*P* < 0.05; **Figure 3L**). Similarly, the SA levels in both wild-type and mutant plants increased after pathogen infection compared to their respective untreated controls; however, the SA levels in the mutant plants were significantly higher than those in the Hm 2–2 plants (*P* < 0.05; **Figure 3M**).

### 3.4 *SlWRKY75* regulates hormone-related defense signaling

**Figure 4:**
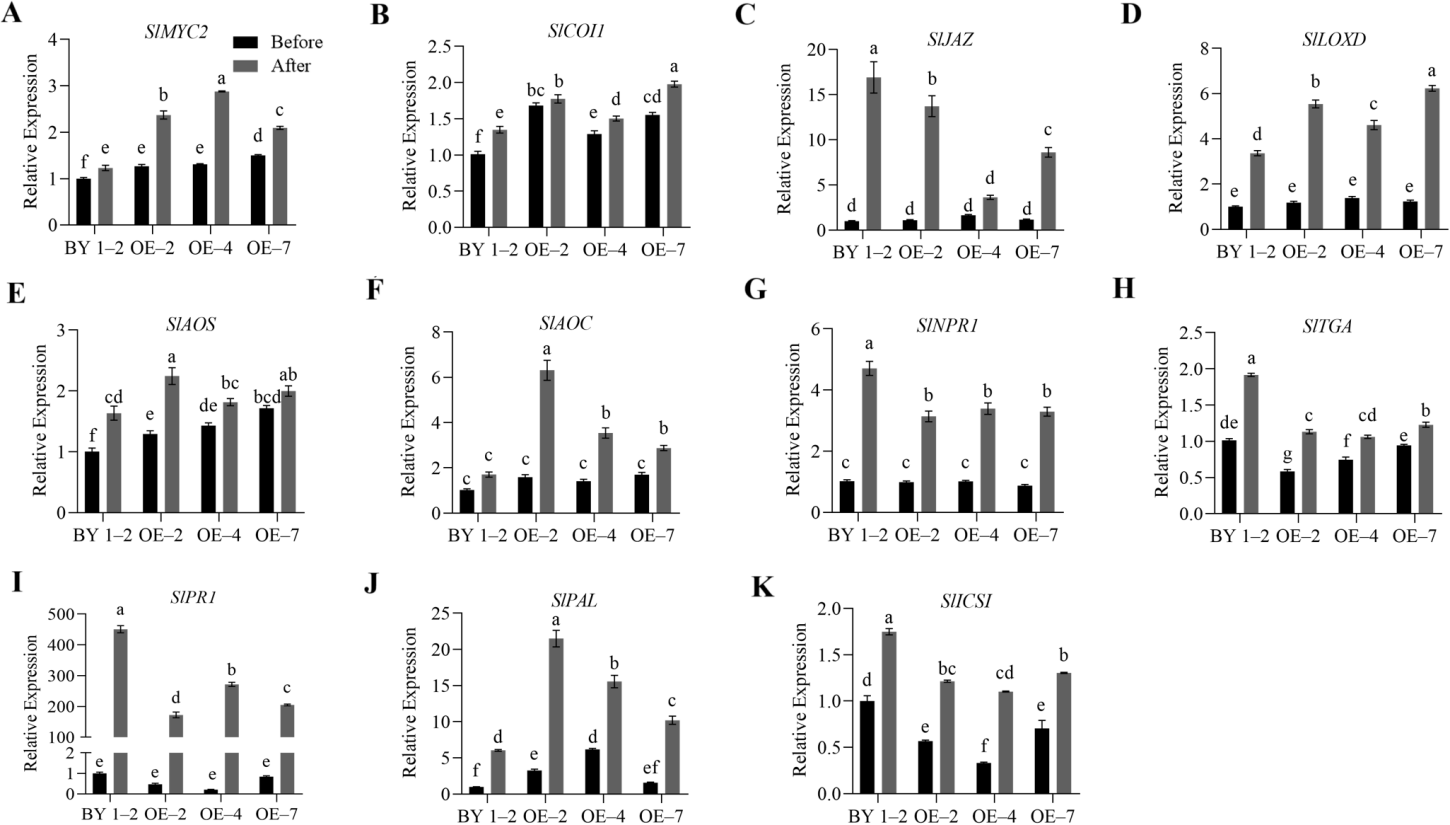
The relative expression levels of the defense signaling genes in wild-type BY 1–2 plants and *SlWRKY75*-overexpressing lines. The expression levels of JA signaling-related genes *SlMYC2*, *SlCOI1*, and *SlJAZ*, and JA biosynthesis-related genes *SlLOXD*, *SlAOS*, and *SlAOC* **(A−F)**, as well as SA signaling-related genes *SlNPR1*, *SlTGA*, and *SlPR1*, and SA biosynthesis-related genes *SlPAL* and *SlICS1* **(G−K)** in BY 1–2 plants and *SlWRKY75*-overexpressing lines (OE–2, OE–4, and OE–7) before and after inoculation with *R. solanacearum* for 48 h. Data are means of three biological replicates ± standard error (SE). Different letters indicate statistically significant differences among groups (Tukey’s honest significant difference test, *P* < 0.05).

**Figure 5:**
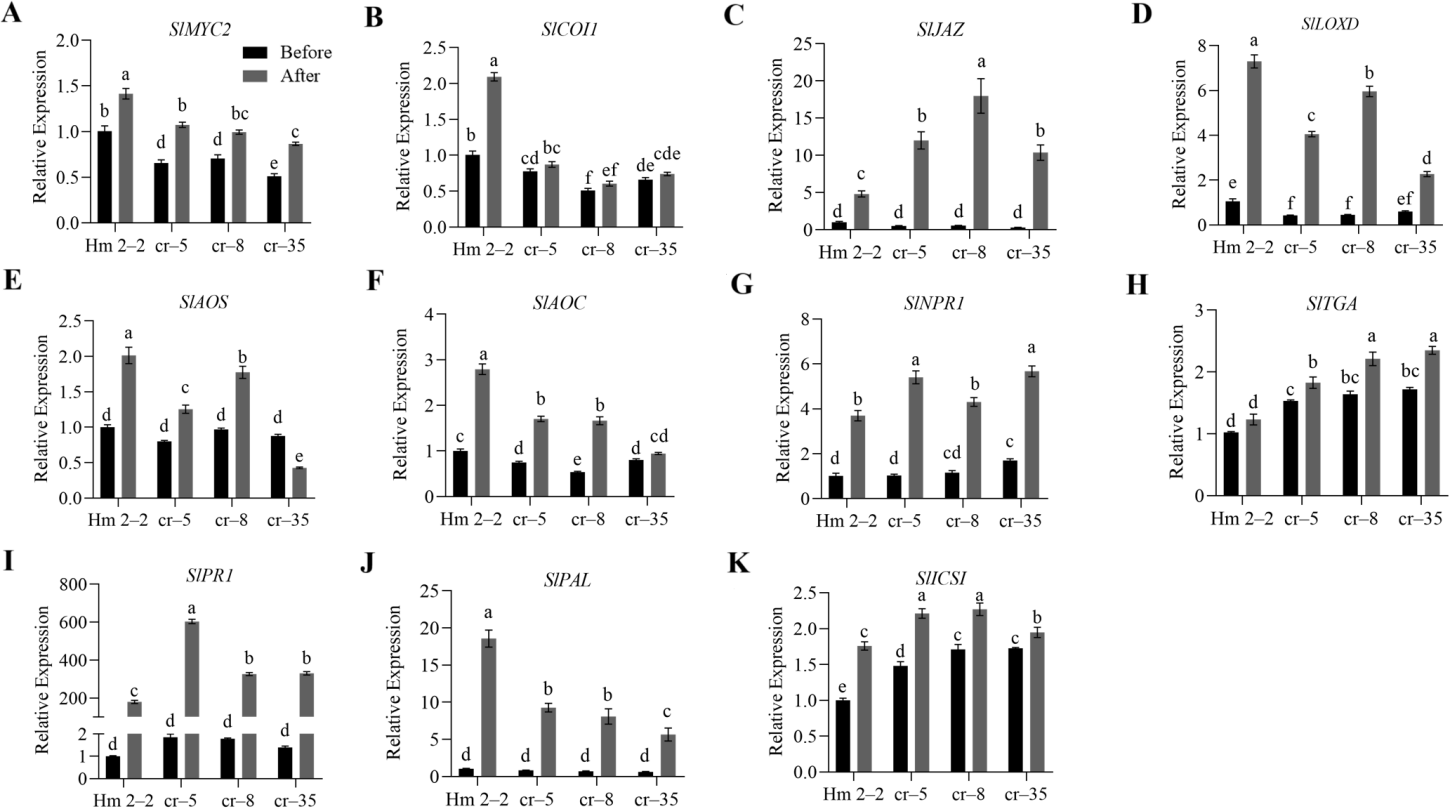
The relative expression levels of the defense signaling genes in wild-type Hm 2–2 plants and *slwrky75* mutant lines. The expression levels of JA signaling-related genes *SlMYC2*, *SlCOI1*, and *SlJAZ*, and JA biosynthesis-related genes *SlLOXD*, *SlAOS*, and *SlAOC* **(A−F)**, as well as SA signaling-related genes *SlNPR1*, *SlTGA*, and *SlPR1*, and SA biosynthesis-related genes *SlPAL* and *SlICS1* **(G−K)** in Hm 2–2 plants and *slwrky75* mutant lines (cr–5, cr–8, and cr–25) before and after inoculation with *R. solanacearum* for 48 h. Data are means of three biological replicates ± standard error (SE). Different letters indicate statistically significant differences among groups (Tukey’s honest significant difference test, *P* < 0.05).

The study detected significant changes in JA and SA levels in the overexpression and mutant lines of tomato after *R. solanacearum* infection. Therefore, to further explore the possible role of *SlWRKY75* in regulating JA and SA signaling in disease resistance, we analyzed the genes related to the JA and SA signaling pathways in the transgenic and mutant plants by qRT-PCR. After inoculation with *R. solanacearum* and incubation for 48 h, the expression of all genes increased. Specifically, the expression levels of JA signaling-related genes *SlMYC2* and *SlCOI1*, JA biosynthesis-related genes *SlLOXD*, *SlAOS*, and *SlAOC*, and SA biosynthesis-related gene *SlPAL* in the transgenic lines (OE–2, OE–4, OE–7) were significantly higher than those in the wild-type BY 1–2 plants (*P* < 0.05; **Figure 4**). Conversely, the expression levels of SA signaling and defense-related genes *SlNPR1*, *SlTGA*, and *SlPR1*, SA biosynthesis-related genes *SlPAL* and *SlICS1*, and JA signaling-related gene *SlJAZ* in the transgenic lines (OE–2, OE–4, OE–7) were significantly lower than those in the BY 1–2 plants (*P* < 0.05; **Figure 4**). Consistent with these observations, after *R. solanacearum* inoculation, the three mutant lines (cr–5, cr–8, cr–35) demonstrated significantly lower expression levels of *SlMYC2*, *SlCOI1*, *SlLOXD*, *SlAOS, SlAOC*, and *SlPAL* than the wild-type Hm 2–2 plants (*P* < 0.05; **Figure 5**). Additionally, the expression levels of *SlNPR1*, *SlTGA*, *SlPR1*, *SlPAL*, *SlICS1*, and *SlJAZ* in the three mutant lines (cr–5, cr–8, cr–35) were significantly higher than those in the wild-type Hm 2–2 plant (*P* < 0.05; **Figure 5**). These results are consistent with the JA and SA levels in the transgenic lines and mutant plants observed after pathogen infection (**Figures 2L, M** and **3L, M**). All these results indicate that *SlWRKY75* regulates tomato’s resistance to *R. solanacearum* through the expression of JA and SA biosynthesis-related and signal response-related genes.

### 3.5 SlWRKY75 directly interacts with the *SlMYC2* promoter

**Figure 6:**
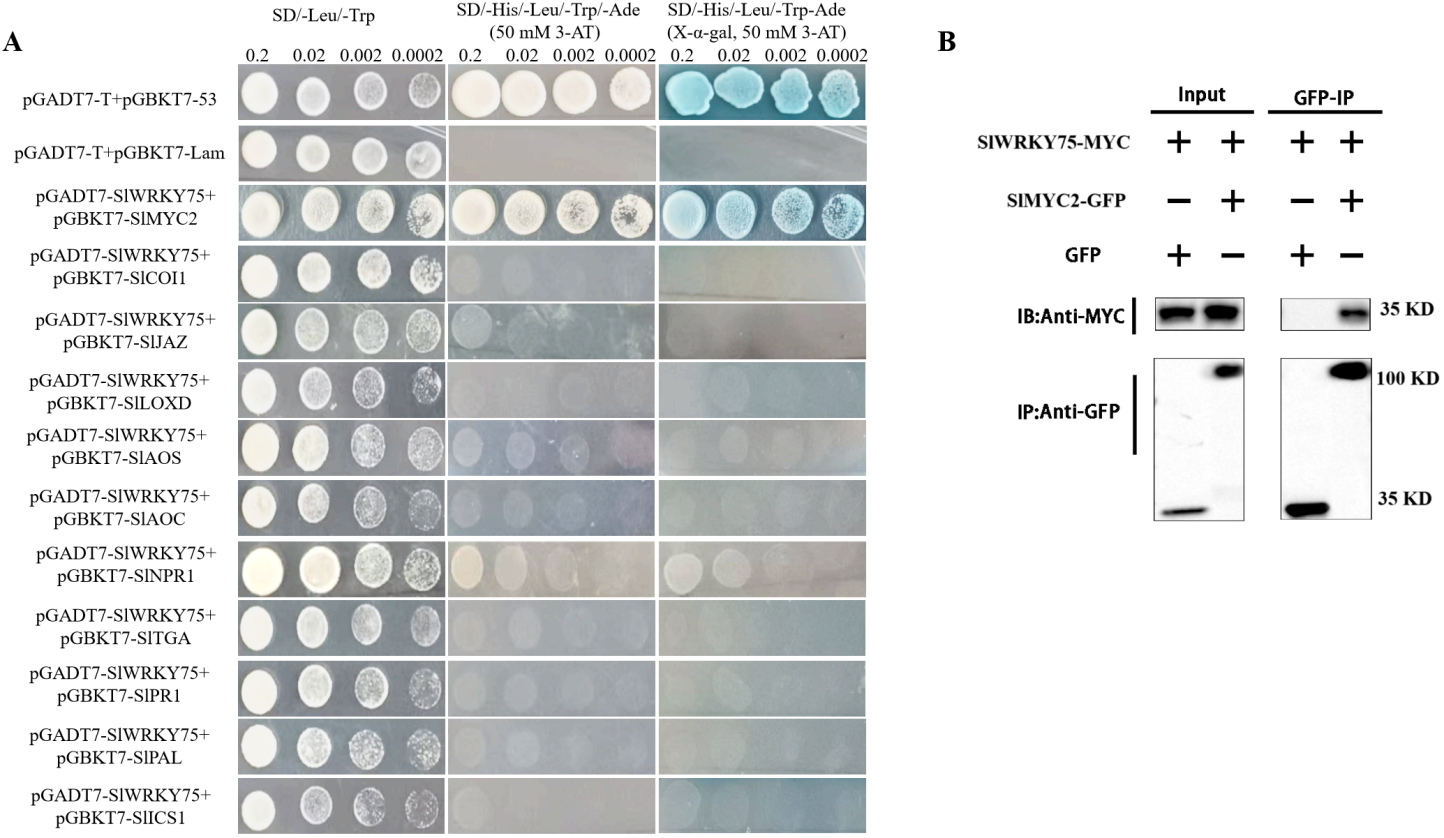
SlWRKY75 interacts with SlMYC2. **(A)** Detection of the interaction between SlWRKY75 and SlMYC2, SlCOI1, SlJAZ, SlLOXD, SlAOS, SlAOC, SlNPR1, SlTGA, SlPR1, SlPAL, SlICS1 by yeast two‐hybrid (Y2H) assays. Yeast cells were cultured on SD/–Leu/–Trp and SD/–His/–Leu/–Trp/–Ade (X-α-gal, 50 mM 3-AT) mediums. Co-transformants containing pGADT7-T with pGBDT7-53 were used as a positive control; co-transformants containing pGADT7-T with pGBDT7-Lam were used as a negative control. Pictures were taken after incubation at 30°C for 3 d **(B)** co-immunoprecipitation (Co-IP) assay for SlWRKY75 and SlMYC2 interaction. MYC-fused SlWRKY75 and GFP-fused SlMYC2 were transiently co-expressed in tobacco leaves. All infected leaves treated with 10 mmol/L MG132 and 20 mmol/L paclobutrazol for 8 h were used for Co-IP. SlMYC2-GFP and GFP tag were immunoprecipitated using anti-GFP mouse antibodies, and co-immunoprecipitated proteins were detected with anti-MYC rabbit antibodies. Protein input for SlWRKY75-MYC proteins in immunoprecipitated complexes was also detected and shown.

Furthermore, the Y2H assay was carried out to investigate whether SlWRKY75 interacts with proteins related to JA and SA synthesis and signal response. Typically, the yeast cells carrying plasmids in different combinations grew well on the SD/-Leu/-Trp medium. However, only the yeast cells containing the plasmid combination pGADT7-SlWRKY75 + pGBKT7-SlMYC2 and the positive control pGADT7-T + pGBKT7-53 grew well on the SD/-Leu/-Trp/-Ade/-His + 3-AT medium and formed blue colonies on the SD/-Leu/-Trp/-Ade/-His + 3-AT + X-α-gal medium. Other plasmid combinations could not grow on these two types of media (**Figure 6A**). These observations indicate that SlWRKY75 interacts with SlMYC2 in yeast cells. Subsequent Co-IP experiment in tobacco leaf tissues confirmed this interaction. The experiment showed that when expressed together with SlMYC2-GFP, SlWRKY75-MYC could be co-immunoprecipitated with the anti-MYC resin, while no co-immunoprecipitation signal was found in the negative control (**Figure 6B**). These results prove that SlWRKY75 interacts with SlMYC2 both *in vitro* and *in vivo*.

**Figure 7:**
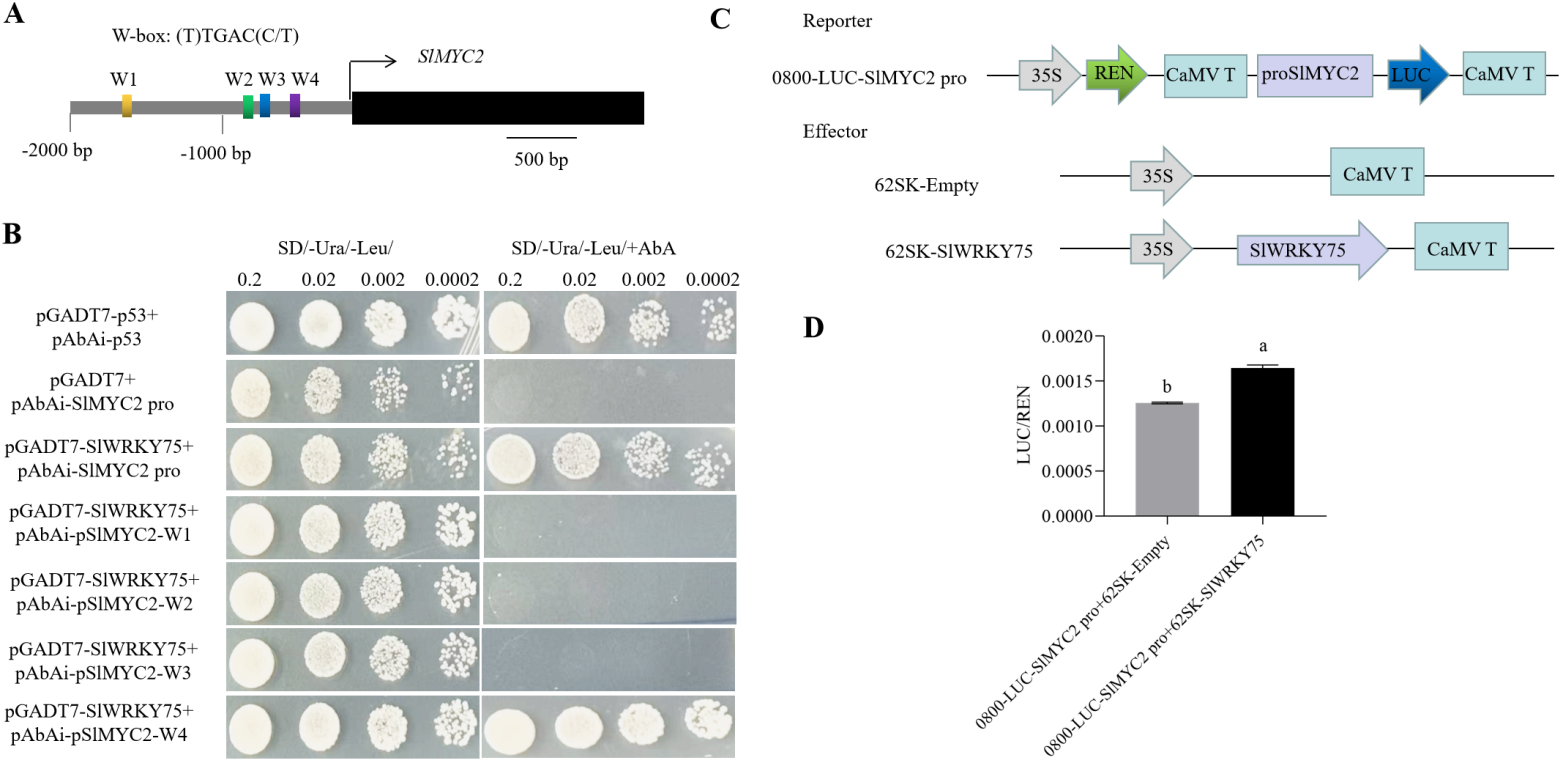
SlWRKY75 directly promotes *SlMYC2* transcriptions by binding to its promoter. **(A)** The promoter region of *SlMYC2* (2000 bp upstream of the translation initiation codon of *SlMYC2*). **(B)** A yeast-one-hybrid (Y1H) assay shows that SlWRKY75 binds to the promoter of *SlMYC2* at the W4 region. Co-transformants containing different constructs (pGADT7-SlWRKY75 with pAbAi-SlMYC2 pro, or pAbAi-pSlMYC2-W1 to W4, respectively) were grown on SD/-Ura/-Leu and SD/-Ura/-Leu mediums plus Aureobasidin A (AbA) for 3 d at 30°C. Co-transformants containing pGADT7-p53 with pAbAi-p53 were used as a positive control; co-transformants containing pGADT7 with pAbAi-SlMYC2 pro were used as a negative control. **(C)** Schematic diagram of the trans-activation assay effector and reporter constructs. The reporter vector carrying the renilla luciferase gene under the control of the 35S promoter and the firefly luciferase reporter gene under the control of the SlMYC2 promoters. **(D)** Luciferase activity analysis using 62SK-SlWRKY75 as effector and 0800-LUC-SlMYC2 pro as reporters. Values of LUC/REN activity ratio are mean ± standard error (SE); n = 3. Different letters indicate statistically significant differences among groups (Tukey’s honest significant difference test, *P* < 0.05).

Bioinformatics analysis revealed four potential W-boxes ((T)TGAC(C/T)) in the promoter region (-2000 bp region upstream) of *SlMYC2* (W1, W2, W3, and W4) (**Figure 7A**). Then, a Y1H experiment was conducted to determine the exact site in the *SlMYC2* promoter where SlWRKY75 interacts or binds. The results showed that all the co-transformed yeast cells could grow normally in the SD/-Ura/-Leu medium. However, when 500 ng/mL AbA was added to the SD/-Ura/-Leu medium, the growth of the pGADT7-p53 + pAbAi-p53 (positive control), pGADT7-SlWRKY75 + pAbAi-SlMYC2 pro, and pGADT7-SlWRKY75 + pAbAi-pSlMYC2-W4 groups were maintained, while those of the pGADT7 + pAbAi-SlMYC2 pro (negative control), pGADT7-SlWRKY75 + pAbAi-pSlMYC2-W1, pGADT7-SlWRKY75 + pAbAi-pSlMYC2-W2, and pGADT7-SlWRKY75 + pAbAi-pSlMYC2-W3 groups were completely inhibited (**Figure 7B**). These observations indicate that SlWRKY75 interacts with the *SlMYC2* promoter at the W4 region. Furthermore, to verify whether SlWRKY75 functions as a transcriptional activator for *SlMYC2* expression, the *SlMYC2* promoter sequence was inserted into the pGreenII0800 vector to generate a reporter, and the *SlWRKY75* CDS was inserted into the pGreenII 62-SK vector to generate an effector (**Figure 7C**). Then, a dual-luciferase-based transcriptional activation assay was performed, and the LUC luminescence intensity and the ratio of LUC activity to REN activity were measured to determine the *SlMYC2* promoter activity. The results showed that the expression level of *SlMYC2* pro::LUC was significantly activated in the presence of SlWRKY75 compared with the control (*P* < 0.0001; **Figure 7D**). This observations indicate that SlWRKY75 exerts a positive transcriptional regulatory effect on *SlMYC2* by directly binding to its promoter, thereby promoting *SlMYC2* expression.

## 4 Discussion

While WRKY transcription factors are known to mediate broad stress responses, their specific roles in tomato resistance to *R. solanacearum* remain largely unexplored. Our previous research showed that *R. solanacearum* infection significantly induced *SlWRKY75* expression, and silencing this gene reduced the resistance of tomato plants (Chen et al., 2023). The present study demonstrated that *SlWRKY75* positively regulates the resistance of tomato to *R. solanacearum*. We found that *SlWRKY75* achieved this response by enhancing antioxidant enzyme and defense enzyme activity, regulating the JA and SA signaling pathways, and maintaining ROS homeostasis. Most importantly, we identified *SlMYC2* as a direct target of SlWRKY75, revealing a novel transcriptional module in JA-mediated immunity against bacterial wilt.

Transcription factors, especially WRKY family members, play crucial roles in plant disease resistance. The observations based on the subcellular localization and transcriptional activation analysis (**Figure 1**) in this study are consistent with the features reported for WRKY proteins of other species (Ma et al., 2025; Wu et al., 2025). In cotton, the pathogen *Verticillium dahliae* significantly induces *GhWRKY70* expression. Overexpression of *GhWRKY70* in *Arabidopsis thaliana* enhanced its resistance to *V. dahliae*, suggesting that *GhWRKY70* functions as a factor positively regulating the response to *V. dahliae* (Zhang et al., 2023b). Besides, transient overexpression of *CpWRKY50* in papaya and heterologous expression of *CpWRKY50* in tomato enhanced the resistance to *Colletotrichum brevisporum*, indicating a positive regulatory effect (Yang et al., 2024b). Studies have also proven the positive regulatory effect of *AtWRKY30* on wheat resistance to *Pseudomonas syringae* pv. *syringae* (Pss) and *Puccinia striiformis* f. sp. *tritici* (Pst) (El-Esawi et al., 2025). Similarly, the present study found that *SlWRKY75* positively regulates the resistance of tomato to *R. solanacearum*. However, in peppers, *CaWRKY20* is known to negatively regulate the resistance to *Colletotrichum scovillei* (Li et al., 2025). These earlier reports and the present study’s findings suggest that WRKY transcription factors positively or negatively regulate plant disease resistance.

Under adverse environmental conditions such as biotic or abiotic stress, a large amount of ROS is produced within the cells, leading to the accumulation of these species and free radicals (Muchira et al., 2021). Plants employ key antioxidant enzymes, such as SOD, CAT, and POD, and the compound MDA to maintain the balance of ROS and slow down the accumulation of free radicals and peroxidation of membrane lipids (Han et al., 2021). The ROS scavenging enzyme SOD catalyzes the dismutation of O_2_^2^ radicals, while CAT and POD remove H_2_O_2_. Besides, MDA content is closely related to the accumulation of ROS and peroxidation of membrane lipids (Liu et al., 2007; Tan et al., 2010). Previous studies have shown that WRKYs can dynamically maintain the steady state balance of ROS-related substances (H_2_O_2_, O_2_^−^, and MDA) by finely regulating the activities of key enzymes such as SOD, POD, and CAT; these processes thereby effectively enhance plant resistance (Zhang et al., 2023b). Cui et al. (2018) found that the overexpression of *SpWRKY3* from *Solanum pimpinellifolium* L3708 enhanced the resistance of tomato plants to *Phytophthora infestans* by upregulating the activities of SOD and POD and reducing MDA, O_2_^−^, and H_2_O_2_ contents. Shu et al. (2021) demonstrated after *B. cinerea* infection, the activities of POD and CAT and the content of O_2_^−^ increased in *SlWRKY46*-overexpressing plants. In our study, after inoculation with *R. solanacearum*, the activities of SOD, POD, and CAT in *SlWRKY75-*overexpressing plants were significantly higher than those in the wild type, while the contents of MDA, H_2_O_2_, and O_2_^−^ accumulated were significantly lower. The opposite trend was observed in *slwrky75* mutants. Additionally, PAL is a key enzyme in plant disease resistance and an important indicator of the plant’s resistance to adverse environments (Shadle et al., 2003). Meanwhile, PPO catalyzes the oxidation of phenolic compounds to form quinones, with direct antibacterial and cytotoxic effects (Li and Steffens, 2002). The *GhWRKY70*-overexpressing cotton plants after inoculation with *Verticillium dahliae* showed significantly increased activities of defense enzymes PAL and PPO, while *GhWRKY70*-silenced plants demonstrated decreased activities (Zhang et al., 2023b). In this study, after inoculation with *R. solanacearum*, the activities of PAL and PPO in *SlWRKY75*-overexpressing plants increased while those in *slwrky75* mutant plants decreased compared to the control plants, respectively (**Figures 2**, **3**), further confirming that *SlWRKY75* positively regulates the resistance to *R. solanacearum* in tomato.

The responses of plants to adverse environmental stresses are often related to hormone-mediated signal transduction pathways. Plant hormones, especially JA and SA, play significant roles in the defense mechanism against pathogens. The JA signaling pathway usually regulates the defense response to necrotrophic pathogens (*Alternaria brassicicola*), while the SA signaling pathway mediates the defense response to biotrophic pathogens (*Hyaloperonospora parasitica*) (Li et al., 2019). A clear antagonistic relationship exists between the JA and SA signaling pathways during the complex regulation of plant responses to external damage. In the complex hormone signaling network, WRKY transcription factors play an important role. Most WRKY transcription factors play regulatory roles at the junctions of these signaling pathways (Wang et al., 2020). In *Nicotiana tabacum*, *NtWRKY50* overexpression significantly increased the SA level but prevented JA generation after pathogen infection (Liu et al., 2017). In *Paeonia lactiflora*, silencing of *PlWRKY65* led to a decrease in JA content and an increase in SA content, which indirectly indicated that *PlWRKY65* promotes the accumulation of JA and inhibits the synthesis of SA (Wang et al., 2020). In cotton, the *GhWRKY53* expression was upregulated after SA and MeJA treatments. Further studies on the content of SA and JA and their related pathway genes showed that the silencing of *GhWRKY53* inhibited the SA pathway and activated the JA pathway, thereby reducing the plant’s resistance to the *B. cinerea* pathogen. These studies proved that *GhWRKY53* positively regulates pathogen resistance via the SA and JA pathways (Li et al., 2023). However, our study found that under *R. solanacearum* infection, the JA content in plants overexpressing *SlWRKY75* was significantly higher than that in the wild-type plants, while the SA content was significantly lower than that in the wild-type plants (**Figure 2**). Knocking out *SlWRKY75* showed the opposite trend (**Figure 3**). These results confirm the antagonistic responses of these two hormones and indicate that *SlWRKY75* positively regulates the JA-mediated signaling pathway and inhibits the SA signaling pathway. This observation suggests that each WRKY protein plays a different role and possesses great potential in stress resistance.

Furthermore, to investigate the role of *SlWRKY75* in resisting *R. solanacearum* invasion, we monitored the relative expression levels of JA and SA signaling and biosynthetic genes in the overexpressing and mutant plants before and after *R. solanacearum* infection. We found that after *R. solanacearum* infection, the expression of JA pathway genes (*SlMYC2*, *SlCOI1*, *SlLOXD*, *SlAOS*, and *SlAOC*) and *SlPAL* in transgenic tomato plants were enhanced, while the expression of SA pathway genes (*SlNPR1*, *SlTGA*, *SlPR1*, and *SlICS1*) and *SlJAZ* were decreased (**Figure 4**). An opposite trend was observed in the mutant plants (**Figure 5**). These results are consistent with the changes in JA and SA contents observed in the overexpressing and mutant plants (**Figures 2**, **3**). Therefore, we speculated that *SlWRKY75* may be related to JA and SA signaling in tomato during pathogen infection. Li et al. (2023) reported that after inoculation with *Verticillium dahliae*, the relative expression levels of SA biosynthetic gene (*GhPAL*) and response gene (*GhPR1*) and the content of SA in *GhWRKY53*-silenced plants decreased, while the relative expression levels of JA biosynthetic gene *GhLOX* and the JA signaling gene *GhPDF1.2* and the content of JA increased. Additionally, we found that SlWRKY75 interacted with SlMYC2 (**Figure 6**), and SlWRKY75 bound to the *SlMYC2* promoter at the W-box, thereby regulating the expression of *SlMYC2* (**Figure 7**). These data indicate that *SlMYC2* is the target gene of SlWRKY75. Our findings present an interesting contrast with the recent report by Wang et al. (2024). Both identified that SlMYC2 can bind to WRKY transcription factors (in this study, SlWRKY75; Wang et al. referred to it as SlWRKY75). However, the SlWRKY75-SlMYC2 module was reported under cold stress, while our SlWRKY75-SlMYC2 module functions in resistance to bacterial wilt. These comparisons suggest that WRKY-MYC2 may be a conserved regulatory partnership in tomatoes for coping with various stresses. Future studies can systematically analyze whether other tomato WRKY members can also directly regulate SlMYC2, thereby mapping out a complete SlWRKYs-SlMYC2 regulatory network. Typically, JAZs (Jasmonate ZIM-domain proteins) are recognized as inhibitors of the JA signaling pathway. When the external environment stimulates plants, a large amount of JA is synthesized, promoting the degradation of JAZ proteins. MYC2, the target gene of JAZ proteins, is released along with the degradation of JAZ proteins, which is turn activate the downstream JA response-related genes (Devoto et al., 2002; Bai et al., 2011; Fernandez-Calvo et al., 2011). In this study, after infection with *R. solanacearum*, the JA content in the overexpressing plants increased, *SlJAZ* expression decreased, and *SlMYC2* expression increased. These changes kept the JA signaling pathway continuously active and enhanced the plant’s disease resistance. However, in the mutant plants, the expression of *SlJAZ* increased while that of *SlMYC2* decreased, subsequently shutting down the JA signaling pathway. These changes reduced the plant’s resistance to *R. solanacearum*. Previous studies have identified two SA biosynthetic pathways in plants: the phenylalanineammonia lyase (PAL) and the isochorismate synthase (ICS) pathway (Wildermuth et al., 2001; Rekhter et al., 2019). Wu et al. (2023) discovered that phenylalanine does not synthesize SA (2-Hydroxybenzoic acid, 2-HBA) but instead synthesizes the isomer of SA, 4-hydroxybenzoic acid (4-HBA). In this study, after infection with *R. solanacearum*, the *SlPAL* expression in the overexpressing plants increased, while the *SlICS1* expression decreased, and the SA content decreased. However, in the mutant plants, the *SlPAL* expression decreased and the *SlICS1* expression increased, and the SA content increased. Therefore, the negative correlation between *SlWRKY75* activity and both *SlICS1* expression and SA accumulation—with overexpression suppressing and mutation enhancing them—suggests that *SlWRKY75* indirectly represses the *SlICS1*-mediated SA biosynthetic pathway. Conversely, *SlWRKY75* activates *SlPAL* expression. As PAL-driven flux is likely channeled toward non-SA metabolites like lignin and flavonids (He et al., 2023b; Yao et al., 2023), this indicates that *SlWRKY75* orchestrates a defense metabolic shift away from SA biosynthesis and toward alternative phenylpropanoid defenses.

## 5 Conclusion

**Figure 8:**
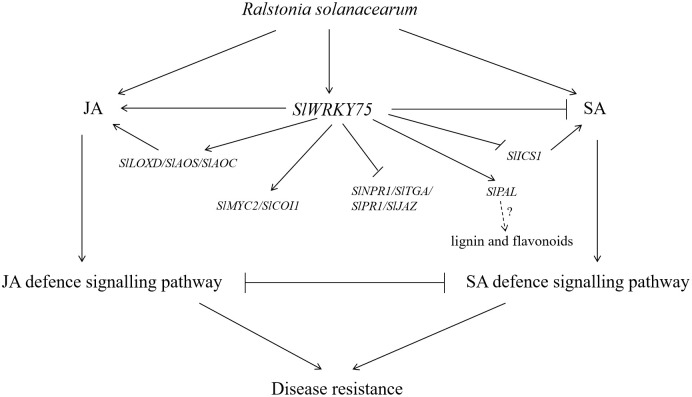
Model diagram of the participation of *SlWRKY75* in the JA and SA regulatory pathway in response to infection with *R. solanacearum.* Solid lines represent the determined regulatory effect, arrows represent the facilitated effect, and short lines represent inhibition. The dotted lines indicate the uncertain regulatory effect. The question mark indicates uncertainty.

The present study proved that *SlWRKY75* plays a positive role in regulating tomato defense against *R. solanacearum*. Overexpression of *SlWRKY75* increased the content of JA (via the activation of the response genes of the JA signaling pathway) but reduced the content of SA (via the inhibition of the response genes of the SA signaling pathway). These changes were found to be accompanied by an increase in the activities of antioxidant enzymes and defense enzymes and a decrease in the contents of MDA, H_2_O_2_, and O_2_^−^,ultimately enhancing its resistance to *R. solanacearum*. *In vitro* assays indicated that SlWRKY75 directly binds to the *SlMYC2* promoter and influences its expression, thereby regulating the JA signaling pathway. These results suggest that *SlWRKY75* participates in a complex network that regulates the response and mediates resistance to *R. solanacearum* by modulating the expression of SA and JA hormone signaling-related genes. As summarized in **Figure 8**, our results demonstrate that SlWRKY75 acts as a positive regulator in tomato resistance against *R. solanacearum*. The proposed model illustrates that *SlWRKY75* enhances the JA signaling pathway by up-regulating the expression of JA synthesis and related signaling genes and directly activating the expression of *SlMYC2*, while it inhibits the SA signaling pathway by down-regulating the expression of SA synthesis and related signaling genes. These findings broaden our understanding of the role of tomato WRKY transcription factors in tomato pathogen resistance.

While this study establishes a crucial role for *SlWRKY75* in resistance to *R. solanacearum*, several intriguing questions remain for future investigation. For instance, whether other WRKY transcription factors function alongside *SlWRKY75*, as functional redundancy and complex interactions are hallmarks of the WRKY family. Given its role in JA-mediated defense established here, *SlWRKY75* may operate in a network with related members. Furthermore, it would be valuable to explore whether *SlWRKY75* also participates in the response to other biotic (e.g., fungal, viral) or abiotic (e.g., high temperature, salinity) stresses, which would define its functional breadth. Elucidating the comprehensive interaction network of *SlWRKY75*, including identifying upstream regulators and downstream targets as well as its potential partners among other transcription factor families, is essential to fully integrate it into the complex signaling web governing plant immunity. Such efforts will provide a more complete picture of the molecular mechanisms underlying *SlWRKY75*-mediated resistance and could inform strategies for breeding disease-resistant crops.

## Data Availability

The original contributions presented in the study are included in the article/**Supplementary Material**. Further inquiries can be directed to the corresponding author.

## Supplementary material

Supplementary Figure 1 Overexpression transgenic plants of the SlWRKY75. **(A)** A schematic map of the pBI121-SlWRKY75 recombinant vector construction. **(B)** Agarose gel electrophoresis detection diagram of PCR. M: DNA Mark DL 2000 bp, WT: wild type BY 1–2 (susceptible to *R. solanacearum*) plants, CK+: pBI121-*SlWRKY75* recombinant plasmid. OE 1–8: *SlWRKY75*-overexpressing plants. **(C)** The relative expression of *SlWRKY75* in BY 1–2 and *SlWRKY75*-overexpressing plants. Data are means of three biological replicates ± standard error (SE). Different letters indicate statistically significant differences among groups (Tukey’s honest significant difference test, *P* < 0.05).

Supplementary Figure 2 CRISPR/Cas9-induced mutation in the *SlWRKY75*. **(A)** A schematic map of the sgRNA targeted sites on the genomic regions of *SlWRKY75*. The intergenic region and introns are shown as lines; the exons are shown as gray boxes. The PAM motifs (NGG) are shown in red. **(B)** Sequencing results of the *SlWRKY75* homozygous mutant lines from the T1 generation. The PAM motif is highlighted in gray, the target sequence is underlined, and the red highlighting dashes indicate deletions. WT: wild type Hm 2–2 plants (resistant to *R. solanacearum*), cr–5, cr–8, cr–35: *slwrky75*-mutant lines. **(C)** The relative expression of *SlWRKY75* in Hm 2–2 and mutant lines. Data are means of three biological replicates ± standard error (SE). Different letters indicate statistically significant differences among groups (Tukey’s honest significant difference test, *P* < 0.05).

## References

[B1] Bai Y. Meng Y. Huang D. Qi Y. Chen M. (2011). Origin and evolutionary analysis of the plant-specific TIFY transcription factor family. Genomics 98, 128–136. doi: 10.1016/j.ygeno.2011.05.002, PMID: 21616136

[B2] Bi Y. Wang H. Yuan X. Yan Y. Li D. Song F. (2023). The NAC transcription factor ONAC083 negatively regulates rice immunity against Magnaporthe oryzae by directly activating transcription of the RING-H2 gene OsRFPH2-6. J. Integr. Plant Biol. 65, 854–875. doi: 10.1111/jipb.13399, PMID: 36308720

[B3] Cao P. Xia L. Li X. Deng M. Zhang Z. Lin X.. (2025). A SlMYB78-regulated bifunctional gene cluster for phenolamide and salicylic acid biosynthesis during tomato domestication, reducing disease resistance. J. Integr. Plant Biol. 67, 1947–1964. doi: 10.1111/jipb.13899, PMID: 40152226 PMC12225020

[B4] Chen H. Shi Y. An L. Yang X. Liu J. Dai Z.. (2024). Overexpression of SlWRKY6 enhances drought tolerance by strengthening antioxidant defense and stomatal closure via ABA signaling in Solanum lycopersicum L. Plant Physiol. Biochem. 213, 108855. doi: 10.1016/j.plaphy.2024.108855, PMID: 38917736

[B5] Chen J. Wang H. Li Y. Pan J. Hu Y. Yu D. (2018). Arabidopsis VQ10 interacts with WRKY8 to modulate basal defense against Botrytis cinerea. J. Integr. Plant Biol. 60, 956–969. doi: 10.1111/jipb.12664, PMID: 29727045

[B6] Chen K. Khan R. A. A. Cao W. Ling M. (2020). Sustainable and ecofriendly approach of managing soil born bacterium Ralstonia solanacearum (Smith) using dried powder of Conyza canadensis. Pathogens 9, 327. doi: 10.3390/pathogens9050327, PMID: 32349319 PMC7281776

[B7] Chen N. Shao Q. Lu Q. Li X. Gao Y. (2022). Transcriptome analysis reveals differential transcription in tomato (Solanum lycopersicum) following inoculation with Ralstonia solanacearum. Sci. Rep. 12, 22137. doi: 10.1038/s41598-022-26693-y, PMID: 36550145 PMC9780229

[B8] Chen N. Wu S. Fu J. Cao B. Lei J. Chen C.. (2016). Overexpression of the eggplant (Solanum melongena) NAC family transcription factor SmNAC suppresses resistance to bacterial wilt. Sci. Rep. 6, 31568. doi: 10.1038/srep31568, PMID: 27528282 PMC4985710

[B9] Chen N. Zhan W. W. Liu X. Y. Shi L. X. Li R. N. Xie R.. (2023). Cloning, expression, and functional analysis of WRKY transcription factor SlWRKY75 in tomato. Acta Agric. Boreali-Sin. 38, 1–10. doi: 10.7668/hbnxb.20194162

[B10] Chen Z. Lin E. Lin X. Liu L. Li W. Feng J.. (2025). Metabolomic profiling of tomato root exudates induced by Ralstonia solanacearum strains of different pathogenicity: screening for metabolites conferring bacterial wilt resistance. J. Microbiol. Biotechnol. 35, e2501033. doi: 10.4014/jmb.2501.01033, PMID: 40443231 PMC12149398

[B11] Cui D. D. (2018). Expression analysis of ARFs and WRKYs in pathogenesis of tomato bacterial wilt, Master’s thesis (China: South China Agricultural University).

[B12] Cui J. Xu P. Meng J. Li J. Jiang N. Luan Y. (2018). Transcriptome signatures of tomato leaf induced by Phytophthora infestans and functional identification of transcription factor SpWRKY3. Theor. Appl. Genet. 131, 787–800. doi: 10.1007/s00122-017-3035-9, PMID: 29234827

[B13] Dang F. Lin J. Li Y. Jiang R. Fang Y. Ding F.. (2023). SlWRKY30 and SlWRKY81 synergistically modulate tomato immunity to Ralstonia solanacearum by directly regulating SlPR-STH2. Hortic. Res. 10, uhad050. doi: 10.1093/hr/uhad050, PMID: 37206055 PMC10189802

[B14] Devoto A. Nieto-Rostro M. Xie D. Ellis C. Harmston R. Patrick E.. (2002). COI1 links jasmonate signalling and fertility to the SCF ubiquitin-ligase complex in Arabidopsis. Plant J. 32, 457–466. doi: 10.1016/j.hpj.2023.05.005, PMID: 12445118

[B15] Dong D. L. Tian Y. Zhang X. M. Duan D. Y. Zhang H. Yang K. Y.. (2024). Overexpression of the transcription factor MdWRKY115 improves drought and osmotic stress tolerance by directly binding to the MdRD22 promoter in apple. Hortic. Plant J. 10 (3), 629–640. doi: 10.1016/j.hpj.2023.05.005

[B16] El-Esawi M. A. Ali H. M. El-Ballat E. M. (2025). AtWRKY30 transcription factor mitigates chromium and salt toxicity and induces resistance against bacterial leaf blight and stripe rust in wheat. Physiol. Plant 177, e70243. doi: 10.1111/ppl.70243, PMID: 40302154

[B17] Eulgem T. Rushton P. J. Robatzek S. Somssich I. E. (2000). The WRKY superfamily of plant transcription factors. Trends Plant Sci. 5, 199–206. doi: 10.1016/s1360-1385(00)01600-9, PMID: 10785665

[B18] Fan X. Chen M. Zhang H. Liu Y. Yang M. Ye C.. (2025). Systematic identification and analysis of WRKY transcription factors reveals the role of MrWRKY14 in Myrica rubra. Front. Plant Sci. 16. doi: 10.3389/fpls.2025.1602750, PMID: 40519594 PMC12162600

[B19] Fang X. Wu H. Huang W. Ma Z. Jia Y. Min Y.. (2024). The WRKY transcription factor ZmWRKY92 binds to GA synthesis-related genes to regulate maize plant height. Plant Physiol. Biochem. 207, 108422. doi: 10.1016/j.plaphy.2024.108422, PMID: 38335889

[B20] Fernández-Calvo P. Chini A. Fernández-Barbero G. Chico J. M. Gimenez-Ibanez S. Geerinck J.. (2011). The Arabidopsis bHLH transcription factors MYC3 and MYC4 are targets of JAZ repressors and act additively with MYC2 in the activation of jasmonate responses. Plant Cell 23, 701–715. doi: 10.1105/tpc.110.080788, PMID: 21335373 PMC3077776

[B21] Fradin E. F. Zhang Z. Juarez Ayala J. C. Castroverde C. D. Nazar R. N. Robb J.. (2009). Genetic dissection of Verticillium wilt resistance mediated by tomato Ve1. Plant Physiol. 150, 320–332. doi: 10.1104/pp.109.136762, PMID: 19321708 PMC2675724

[B22] Gowtham H. G. Murali M. Shilpa N. Amruthesh K. N. Abdul. G. Sarjiya A.. (2024). Harnessing abiotic elicitors to bolster plant’s resistance against bacterial pathogens. Plant Stress 11, 100371. doi: 10.1016/j.stress.2024.100371

[B23] Han D. H. Wang E. J. Zhang Y. (2021). Effects of REE fertilizer on physiological characteristics of Astragalus membranaceus seedling under drought stress. Chin. Wild Plant Resour. 40, 33–37.

[B24] He F. Kong D. Feng Z. Xu Y. Yuan Q. Liu D.. (2023a). Genome-wide identification of the NPR1-like gene family in Solanum tuberosum and functional characterization of StNPR1 in resistance to Ralstonia solanacearum. Genes (Basel) 14, 1170. doi: 10.3390/genes14061170, PMID: 37372350 PMC10298024

[B25] He S. Xu X. Gao Q. Huang C. Luo Z. Liu P.. (2023b). NtERF4 promotes the biosynthesis of chlorogenic acid and flavonoids by targeting PAL genes in Nicotiana tabacum. Planta 259, 31. doi: 10.1007/s00425-023-04301-1, PMID: 38150094

[B26] Hou S. Tsuda K. (2022). Salicylic acid and jasmonic acid crosstalk in plant immunity. Essays Biochem. 66, 647–656. doi: 10.1042/EBC20210090, PMID: 35698792

[B27] Ijaz M. Lv L. Ahmed T. Noman M. Manan A. Ijaz R.. (2024). Immunomodulating melatonin-decorated silica nanoparticles suppress bacterial wilt (Ralstonia solanacearum) in tomato (Solanum lycopersicum L.) through fine-tuning of oxidative signaling and rhizosphere bacterial community. J. Nanobiotechnology 22, 617. doi: 10.1186/s12951-024-02910-w, PMID: 39395991 PMC11470696

[B28] Jia S. Wang Y. Zhang G. Yan Z. Cai Q. (2020). Strawberry FaWRKY25 transcription factor negatively regulated the resistance of strawberry fruits to Botrytis cinerea. Genes (Basel) 12, 56. doi: 10.3390/genes12010056, PMID: 33396436 PMC7824073

[B29] Jiang X. Liu X. Chen B. Zhang X. Wang Y. Wang T. (2025). Jasmonic acid-mediated cell wall biosynthesis pathway involved in pepper (Capsicum annuum) defense response to Ralstonia solanacearum. BMC Plant Biol. 25, 804. doi: 10.1186/s12870-025-06784-4, PMID: 40604413 PMC12218943

[B30] Jiang Y. Zheng W. Li J. Liu P. Zhong K. Jin P.. (2021). NbWRKY40 positively regulates the response of Nicotiana benthamiana to tomato mosaic virus via salicylic acid signaling. Front. Plant Sci. 11. doi: 10.3389/fpls.2020.603518, PMID: 33552099 PMC7857026

[B31] Kim B. S. French E. Caldwell D. Harrington E. J. Iyer-Pascuzzi A. S. (2016). Bacterial wilt disease: host resistance and pathogen virulence mechanisms. Physiol. Mol. Plant Pathol. 95, 37–43. doi: 10.1016/j.pmpp.2016.02.007

[B32] Kumar A. Sichov N. Bucki P. Miyara S. B. (2023). SlWRKY16 and SlWRKY31 of tomato, negative regulators of plant defense, involved in susceptibility activation following root-knot nematode Meloidogyne javanica infection. Sci. Rep. 13, 14592. doi: 10.1038/s41598-023-40557-z, PMID: 37669955 PMC10480479

[B33] Li Y. Chen H. Wang Y. Zhu J. Zhang X. Sun J.. (2023). Function analysis of GhWRKY53 regulating cotton resistance to Verticillium wilt by JA and SA signaling pathways. Front. Plant Sci. 14. doi: 10.3389/fpls.2023.1203695, PMID: 37332701 PMC10272532

[B34] Li N. Han X. Feng D. Yuan D. Huang L. J. (2019). Signaling crosstalk between salicylic acid and ethylene/jasmonate in plant defense: do we understand what they are whispering. Int. J. Mol. Sci. 20, 671. doi: 10.3390/ijms20030671, PMID: 30720746 PMC6387439

[B35] Li Y. Ma X. Xiao L. D. Yu Y. N. Gong Z. H. (2025). CaWRKY20 negatively regulates plant resistance to Colletotrichum scovillei in pepper. Plant Cell Environ. 48, 1514–1534. doi: 10.1111/pce.15205, PMID: 39462903

[B36] Li L. Steffens J. (2002). Overexpression of polyphenol oxidase in transgenic tomato plants results in enhanced bacterial disease resistance. Planta 215, 239–247. doi: 10.1007/s00425-002-0750-4, PMID: 12029473

[B37] Liu H. Y. Liang Z. S. Liu S. M. Dong J. E. (2007). Effect of progressive drying and rewatering on protective enzyme activities and osmoregulatory molecules in leaves of Eucommia ulmoides seeding. J. Northwest Forestry Univ. 22, 55–59.

[B38] Liu Q. Liu Y. Tang Y. Chen J. Ding W. (2017). Overexpression of NtWRKY50 increases resistance to Ralstonia solanacearum and alters salicylic acid and jasmonic acid production in tobacco. Front. Plant Sci. 8. doi: 10.3389/fpls.2017.01710, PMID: 29075272 PMC5641554

[B39] Liu X. Shang C. Duan P. Yang J. Wang J. Sui D.. (2025). The SlWRKY42-SlMYC2 module synergistically enhances tomato saline-alkali tolerance by activating the jasmonic acid signaling and spermidine biosynthesis pathway. J. Integr. Plant Biol. 67, 1254–1273. doi: 10.1111/jipb.13839, PMID: 39873954

[B40] Liu F. Xi M. Liu T. Wu X. Ju L. Wang D. (2024). The central role of transcription factors in bridging biotic and abiotic stress responses for plants’ resilience. New Crops 1, 100005. doi: 10.1016/j.ncrops.2023.11.003

[B41] Livak K. J. Schmittgen T. D. (2001). Analysis of relative gene expression data using real-time quantitative PCR and the 2 (-Delta Delta C(T)) method. Methods 25, 402–408. doi: 10.1006/meth.2001.1262, PMID: 11846609

[B42] López-Galiano M. J. González-Hernández A. I. Crespo-Salvador O. Rausell C. Real M. D. Escamilla M.. (2018). Epigenetic regulation of the expression of WRKY75 transcription factor in response to biotic and abiotic stresses in Solanaceae plants. Plant Cell Rep. 37, 167–176. doi: 10.1007/s00299-017-2219-8, PMID: 29079899

[B43] Luo S. D. (2024). Identification of the tomato GEF gene family and the role of SlHSFA4b-SlWRKYs interactions in response to Ralstonia solanacearum infestation. Master’s thesis. Jiangsu University, China.

[B44] Luo L. Wang Y. Qiu L. Han X. Zhu Y. Liu L.. (2023). MYC2: A master switch for plant physiological processes and specialized metabolite synthesis. Int. J. Mol. Sci. 24, 3511. doi: 10.3390/ijms24043511, PMID: 36834921 PMC9963318

[B45] Ma Z. Jia Y. Min Y. Fang X. Yan H. Ma Q.. (2025). Maize ZmWRKY71 gene positively regulates drought tolerance through reactive oxygen species homeostasis. Plant Physiol. Biochem. 219, 109399. doi: 10.1016/j.plaphy.2024.109399, PMID: 39689610

[B46] Mansfield J. Genin S. Magori S. Citovsky V. Sriariyanum M. Ronald P.. (2012). Top 10 plant pathogenic bacteria in molecular plant pathology. Mol. Plant Pathol. 13, 614–629. doi: 10.1111/j.1364-3703.2012.00804.x, PMID: 22672649 PMC6638704

[B47] Meshram S. Adhikari T. B. (2024). Microbiome-mediated strategies to manage major soil-borne diseases of tomato. Plants (Basel) 13, 364. doi: 10.3390/plants13030364, PMID: 38337897 PMC10856849

[B48] Muchira N. Ngugi K. Wamalwa L. N. Avosa M. Chepkorir W. Manyasa E.. (2021). Genotypic variation in cultivated and wild sorghum genotypes in response to Striga hermonthica infestation. Front. Plant Sci. 12. doi: 10.3389/fpls.2021.671984, PMID: 34305972 PMC8296141

[B49] Ottaviani L. Lefeuvre R. Montes E. Widiez T. Giorni P. Mithöfer A.. (2025). A loss-of-function of ZmWRKY125 induced by CRISPR/Cas9 improves resistance against Fusarium verticillioides in maize kernels. Plant Cell Rep. 44, 144. doi: 10.1007/s00299-025-03544-4, PMID: 40528015

[B50] Peeters N. Guidot A. Vailleau F. Valls M. (2013). Ralstonia solanacearum, a widespread bacterial plant pathogen in the post-genomic era. Mol. Plant Pathol. 14, 651–662. doi: 10.1111/mpp.12038, PMID: 23718203 PMC6638647

[B51] Peng L. Chao D. Sun X. Li L. Yu J. Li Z.. (2025). OsWRKY18, a WRKY transcription factor, is involved in rice salt tolerance. Plant Cell Physiol. 5, pcaf063. doi: 10.1093/pcp/pcaf063, PMID: 40470919

[B52] Pieterse C. M. van der Does D. Zamioudis C. Leon-Reyes A. Van Wees S. C. (2012). Hormonal modulation of plant immunity. Annu. Rev. Cell Dev. Biol. 28, 489–521. doi: 10.1146/annurev-cellbio-092910-154055, PMID: 22559264

[B53] Rajan A. Kumar S. Sunil C. K. Radhakrishnan M. Rawson A. (2022). Recent advances in the utilization of industrial byproducts and wastes generated at different stages of tomato processing: status report. J. Food Process. Preserv. 46, e17063. doi: 10.1111/jfpp.17063

[B54] Rekhter D. Lüdke D. Ding Y. Feussner K. Zienkiewicz K. Lipka V.. (2019). Isochorismate-derived biosynthesis of the plant stress hormone salicylic acid. Science 365 6452), 498–502. doi: 10.1126/science.aaw1720, PMID: 31371615

[B55] Rushton P. J. Somssich I. E. Ringler P. Shen Q. J. (2010). WRKY transcription factors. Trends Plant Sci. 15, 247–258. doi: 10.1016/j.tplants.2010.02.006, PMID: 20304701

[B56] Shadle G. L. Wesley S. V. Korth K. L. Chen F. Lamb C. Dixon R. A. (2003). Phenylpropanoid compounds and disease resistance in transgenic tobacco with altered expression of L-phenylalanine ammonia-lyase. Phytochemistry 64, 153–161. doi: 10.1016/s0031-9422(03)00151-1, PMID: 12946414

[B57] Shi L. Fan Y. Yang Y. Yan S. Qiu Z. Liu Z.. (2024). CaWRKY22b plays a positive role in the regulation of pepper resistance to Ralstonia solanacearum in a manner associated with jasmonic acid signaling. Plants (Basel) 13, 2081. doi: 10.3390/plants13152081, PMID: 39124199 PMC11314181

[B58] Shu P. Zhang S. Li Y. Wang X. Yao L. Sheng J.. (2021). Over-expression of SlWRKY46 in tomato plants increases susceptibility to Botrytis cinerea by modulating ROS homeostasis and SA and JA signaling pathways. Plant Physiol. Biochem. 166, 1–9. doi: 10.1016/j.plaphy.2021.05.021, PMID: 34087740

[B59] Shui D. Sun J. Xiong Z. Zhang S. Shi J. (2024). Comparative identification of WRKY transcription factors and transcriptional response to Ralstonia solanacearum in tomato. Gene 912, 148384. doi: 10.1016/j.gene.2024.148384, PMID: 38493971

[B60] Song H. Cao Y. Zhao L. Zhang J. Li S. (2023). Review: WRKY Transcription factors: understanding the functional divergence. Plant Sci. 334, 111770. doi: 10.1016/j.plantsci.2023.111770, PMID: 37321304

[B61] Su M. Yang Y. Lin C. Liu W. Chen X. (2025). WRKY transcription factor MdWRKY71 regulates flowering time in apple. Plant Mol. Biol. 115, 32. doi: 10.1007/s11103-024-01544-8, PMID: 39945922

[B62] Tan Y. A. Bai L. X. Xiao L. B. Wei S. Y. Zhao H. X. (2010). Herbivore stress by Lygus lucorum inducing protective enzyme activity and MDA content on different cotton varieties. Cotton Sci. 22, 479–485. doi: 10.11963/cs100516

[B63] Tang Y. Liu Q. Liu Y. Zhang L. Ding W. (2017). Overexpression of NtPR-Q up-regulates multiple defense-related genes in Nicotiana tabacum and enhances plant resistance to Ralstonia solanacearum. Front. Plant Sci. 8. doi: 10.3389/fpls.2017.01963, PMID: 29201034 PMC5696355

[B64] Wang L. Chen H. Chen G. Luo G. Shen X. Ouyang B.. (2024). Transcription factor SlWRKY50 enhances cold tolerance in tomato by activating the jasmonic acid signaling. Plant Physiol. 194, 1075–1090. doi: 10.1093/plphys/kiad578, PMID: 37935624

[B65] Wang H. Gong W. Wang Y. Ma Q. (2023). Contribution of a WRKY transcription factor, ShWRKY81, to powdery mildew resistance in wild tomato. Int. J. Mol. Sci. 24, 2583. doi: 10.3390/ijms24032583, PMID: 36768909 PMC9917159

[B66] Wang X. Li J. Guo J. Qiao Q. Guo X. Ma Y. (2020). The WRKY transcription factor PlWRKY65 enhances the resistance of Paeonia lactiflora (herbaceous peony) to Alternaria tenuissima. Hortic. Res. 7, 57. doi: 10.1038/s41438-020-0267-7, PMID: 32284869 PMC7113260

[B67] Wang B. Wei J. Song N. Wang N. Zhao J. Kang Z. (2018). A novel wheat NAC transcription factor, TaNAC30, negatively regulates resistance of wheat to stripe rust. J. Integr. Plant Biol. 60, 432–443. doi: 10.1111/jipb.12627, PMID: 29251427

[B68] Wang Y. Yan M. Wang A. Ma X. Tian W. Liu Y.. (2025). Plants accumulate abscisic acid after Ralstonia solanacearum infection for enhanced dehydration tolerance and plant resistance. Front. Plant Sci. 16. doi: 10.3389/fpls.2025.1566215, PMID: 40538869 PMC12176866

[B69] Wildermuth M. C. Dewdney J. Wu G. Ausubel F. M. (2001). Isochorismate synthase is required to synthesize salicylic acid for plant defence. Nature 414, 562–565. doi: 10.1038/35107108, PMID: 11734859

[B70] Wu J. Li M. Wang W. Su Y. Li J. Gong J.. (2025). Identification and functional characterization of AsWRKY9, a WRKY transcription factor modulating alliin biosynthesis in garlic (Allium sativum L.). BMC Biol. 23, 14. doi: 10.1186/s12915-025-02116-y, PMID: 39806468 PMC11731438

[B71] Wu J. Zhu W. Zhao Q. (2023). Salicylic acid biosynthesis is not from phenylalanine in Arabidopsis. J. Integr. Plant Biol. 65, 881–887. doi: 10.1111/jipb.13410, PMID: 36377737

[B72] Xiao Z. Yang W. Yang A. Deng L. Geng R. Xiang H.. (2024). CRISPR/Cas9-mediated knockout of NtMYC2a gene involved in resistance to bacterial wilt in tobacco. Gene 927, 148622. doi: 10.1016/j.gene.2024.148622, PMID: 38878988

[B73] Xing H. L. Dong L. Wang Z. P. Zhang H. Y. Han C. Y. Liu B.. (2014). A CRISPR/Cas9 toolkit for multiplex genome editing in plants. BMC Plant Biol. 14, 327. doi: 10.1186/s12870-014-0327-y, PMID: 25432517 PMC4262988

[B74] Yadav M. Dwibedi V. Sharma S. George N. (2022). Biogenic silica nanoparticles from agro-waste: properties, mechanism of extraction and applications in environmental sustainability. J. Environ. Chem. Eng. 10, 108550. doi: 10.1016/j.jece.2022.108550

[B75] Yan S. Chen N. Huang Z. Li D. Zhi J. Yu B.. (2020). Anthocyanin fruit encodes an R2R3-MYB transcription factor, SlAN2-like, activating the transcription of SlMYBATV to fine-tune anthocyanin content in tomato fruit. New Phytol. 225, 2048–2063. doi: 10.1111/nph.20296, PMID: 31625612

[B76] Yang M. Wang Y. Chen C. Xin X. Dai S. Meng C.. (2024a). Transcription factor WRKY75 maintains auxin homeostasis to promote tomato defense against Pseudomonas syringae. Plant Physiol. 195, 1053–1068. doi: 10.1093/plphys/kiae025, PMID: 38245840

[B77] Yang M. Zhou C. Kuang R. Wu X. Liu C. He H.. (2024b). Transcription factor CpWRKY50 enhances anthracnose eesistance by promoting jasmonic acid signaling in papaya. Plant Physiol. 196, 2856–2870. doi: 10.1093/plphys/kiae479, PMID: 39250752

[B78] Yao X. Liang X. Chen Q. Liu Y. Wu C. Wu M.. (2023). MePAL6 regulates lignin accumulation to shape cassava resistance against two-spotted spider mite. Front. Plant Sci. 13. doi: 10.3389/fpls.2022, PMID: 36684737 PMC9853075

[B79] Zhang S. Dong L. Zhang X. Fu X. Zhao L. Wu L.. (2023b). The transcription factor GhWRKY70 from gossypium hirsutum enhances resistance to verticillium wilt via the jasmonic acid pathway. BMC Plant Biol. 23, 141. doi: 10.1186/s12870-023-04141-x, PMID: 36915047 PMC10012446

[B80] Zhang W. Fu Y. Li M. Zhang S. Zhang Y. Liu X.. (2025b). SlWRKY75 functions as a differential regulator to enhance drought tolerance in tomato (Solanum lycopersicum L.). Plant Physiol. Biochem. 227, 110189. doi: 10.1016/j.plaphy.2025.110189, PMID: 40578131

[B81] Zhang L. L. Gong R. Cui Y. L. Zhong X. H. Li Y. Li R. H.. (2025a). Effect analysis of SmWRKY30 in eggplant resistance to Ralstonia solanacearum by virus induced gene silencing (VIGS). Sci. Agric. Sin. 58, 548–563. doi: 10.3864/j.issn.0578-1752.2025.03.011

[B82] Zhang L. Xing L. Dai J. Li Z. Zhang A. Wang T.. (2024). Overexpression of a grape WRKY transcription factor VhWRKY44 improves the resistance to cold and salt of Arabidopsis thaliana. Int. J. Mol. Sci. 25, 7437. doi: 10.3390/ijms25137437, PMID: 39000546 PMC11242199

[B83] Zhang H. Xu Y. Huang Y. Xiong X. Wu X. Yuan G.. (2023a). Tn-seq identifies Ralstonia solanacearum genes required for tolerance of plant immunity induced by exogenous salicylic acid. Mol. Plant Pathol. 24, 536–548. doi: 10.1111/mpp.13321, PMID: 36912695 PMC10189763

[B84] Zhao Y. Q. Sun C. Hu K. D. Yu Y. Liu Z. Song Y. C.. (2025). A transcription factor SlWRKY71 activated the H_2_S generating enzyme SlDCD1 enhancing the response to Pseudomonas syringae pv DC3000 in tomato leaves. New Phytol. 246, 262–279. doi: 10.1111/nph.20431, PMID: 39887348

[B85] Zhou X. Sun Z. Huang Y. He D. Lu L. Wei M.. (2025). WRKY45 positively regulates salinity and osmotic stress responses in Arabidopsis. Plant Physiol. Biochem. 219, 109408. doi: 10.1016/j.plaphy.2024.109408, PMID: 39721186

[B86] Zhu X. Zhao Y. Shi C. M. Xu G. Wang N. Zuo S.. (2024). Antagonistic control of rice immunity against distinct pathogens by the two transcription modules via salicylic acid and jasmonic acid pathways. Dev. Cell 59, 1609–1622.e4. doi: 10.1016/j.devcel.2024.03.033, PMID: 38640925

