# Supplementary figures and images for: A WRKY transcription factor, SlWRKY75, positively regulates tomato (Solanum lycopersicum L.) resistance to Ralstonia solanacearum
Source: Front Plant Sci. 2025 Oct 30;16:1704937. doi: 10.3389/fpls.2025.1704937 (PMC12611961; doi:10.3389/fpls.2025.1704937)

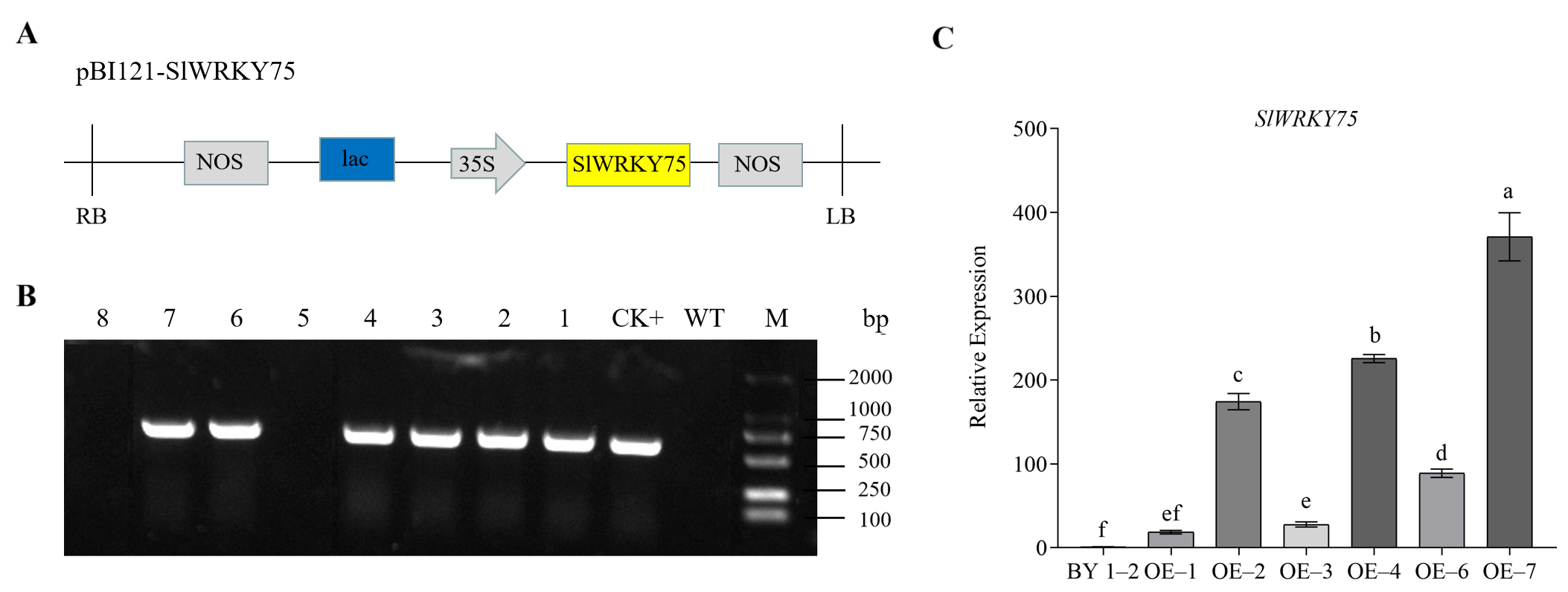

Supplement: Supplementary Figure 1 — Overexpression transgenic plants of the SlWRKY75. (A) A schematic map of the pBI121-SlWRKY75 recombinant vector construction. (B) Agarose gel electrophoresis detection diagram of PCR. M: DNA Mark DL 2000 bp, WT: wild type BY 1–2 (susceptible to R. solanacearum) plants, CK+: pBI121-SlWRKY75 recombinant plasmid. OE 1–8: SlWRKY75-overexpressing plants. (C) The relative expression of SlWRKY75 in BY 1–2 and SlWRKY75-overexpressing plants. Data are means of three biological replicates ± standard error (SE). Different letters indicate statistically significant differences among groups (Tukey’s honest significant difference test, P < 0.05). [file Image1.tif]

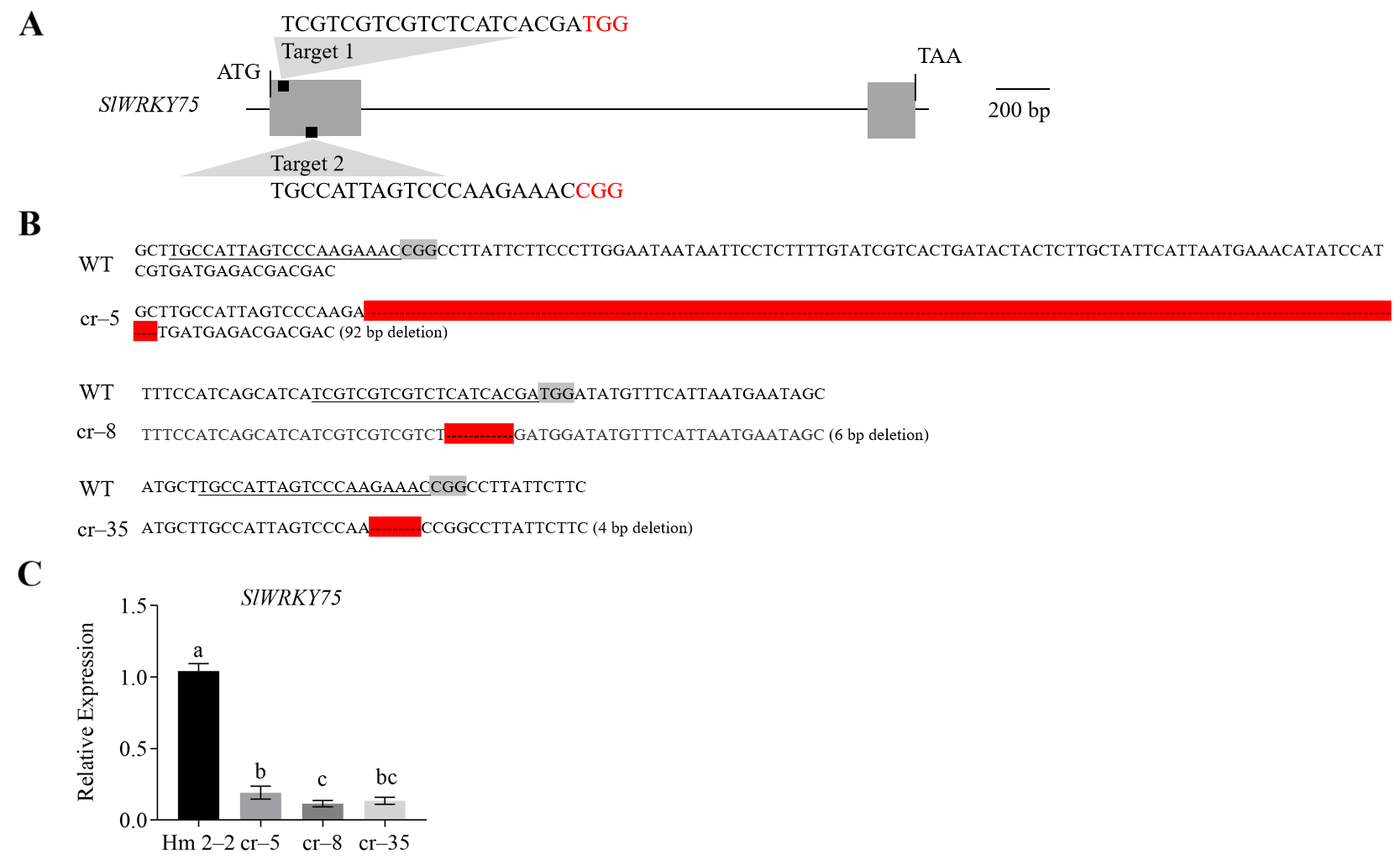

Supplement: Supplementary Figure 2 — CRISPR/Cas9-induced mutation in the SlWRKY75. (A) A schematic map of the sgRNA targeted sites on the genomic regions of SlWRKY75. The intergenic region and introns are shown as lines; the exons are shown as gray boxes. The PAM motifs (NGG) are shown in red. (B) Sequencing results of the SlWRKY75 homozygous mutant lines from the T1 generation. The PAM motif is highlighted in gray, the target sequence is underlined, and the red highlighting dashes indicate deletions. WT: wild type Hm 2–2 plants (resistant to R. solanacearum), cr–5, cr–8, cr–35: slwrky75-mutant lines. (C) The relative expression of SlWRKY75 in Hm 2–2 and mutant lines. Data are means of three biological replicates ± standard error (SE). Different letters indicate statistically significant differences among groups (Tukey’s honest significant difference test, P < 0.05). [file Image2.tif]
