# Supplementary material for: A WRKY transcription factor, SlWRKY75, positively regulates tomato (Solanum lycopersicum L.) resistance to Ralstonia solanacearum
Source: Front Plant Sci. 2025 Oct 30;16:1704937. doi: 10.3389/fpls.2025.1704937 (PMC12611961; doi:10.3389/fpls.2025.1704937)
Supplement: Supplementary file 3 [file Table1.doc]

Table S1. Primers used for vector construction

| Primer | Sequence (5′－3′) |
| --- | --- |
| Sub-*SlWRKY75*-F  Sub-*SlWRKY75*-R  BD-SlWRKY75-F  BD-SlWRKY75-R  pBI121-SlWRKY75-F  pBI121-SlWRKY75-R  Jiance-pBI121-F  Jiance-pBI121-R  CRISPR/Cas9-SlWRKY75-F  CRISPR/Cas9-SlWRKY75-R  Jiance-CRISPR/Cas9-F  Jiance-CRISPR/Cas9-R  AD-SlWRKY75-F  AD-SlWRKY75-R  BD-SlNPR1-F  BD-SlNPR1-R  BD-SlTGA-F  BD-SlTGA-R  BD-SlPR1-F  BD-SlPR1-R  BD-SlPAL-F  BD-SlPAL-R  BD-SlICS1-F  BD-SlICS1-R  BD-SlMYC2-F  BD-SlMYC2-R  BD-SlLOXD-F  BD-SlLOXD-R  BD-SlAOS-F  BD-SlAOS-R  BD-SlAOC-F  BD-SlAOC-R  BD-SlCOI1-F  BD-SlCOI1-R  BD-SlJAZ-1F  BD-SlJAZ-1R  SlMYC2-GFP-F  SlMYC2-GFP-R  SlWRKY75-MYC-F  SlWRKY75-MYC-R  SlMYC2 pro-F  SlMYC2 pro-R  AbAi-SlMYC2 pro-F  AbAi-SlMYC2 pro-R  AbAi-pMYC2-W1-F  AbAi-pMYC2-W1-R  AbAi-pMYC2-W2-F  AbAi-pMYC2-W2-R  AbAi-pMYC2-W3-F  AbAi-pMYC2-W3-R  AbAi-pMYC2-W4-F  AbAi-pMYC2-W4-R   1. SK-SlWRKY75-F   62-SK-SlWRKY75-R  0800-proSlMYC2-F  0800-proSlMYC2-R | CGCGTCGACATGGAGAATTATGCAACAATATTT  CGCGGATCCAAAGGAATTATAGATTTGCATTTG  CCGGAATTCATGGAGAATTATGCAACAATATTT  CGCGGATCCTTAAAAGGAATTATAGATTTGCAT  CGCGGATCCATGGAGAATTATGCAACAATATTT  CGAGCTCTTAAAAGGAATTATAGATTTGCAT  ACGCACAATCCCACTATCCTTCGCA  GACGGCCAGTGAATTCCCGATCTAG  ATATATGGTCTCGTTTGCGTCGTCGTCTCATCACGAGTTTTAGAGCTAGAAATAG  ATTATTGGTCTCGAAACTGCTTGCCATTAGTCCCAACCAAACTACACTGTTAGATTC  ACGTCTCCACTTTTACATTCTCAG  CGGACGGAGGGAGTAGTAATTAAG  GCCATGGAGGCCAGTGAATTCATGGAGAATTATGCAACAATATTTCC  CAGCTCGAGCTCGATGGATCCTTAAAAGGAATTATAGATTTGCATTTGA  ATGGCCATGGAGGCCGAATTCATGGATAGTAGAACTGCTTTTTCGG  CCGCTGCAGGTCGACGGATCCCTATTTCCTAAATGGGAGATTATTGGG  ATGGCCATGGAGGCCGAATTCATGGCTGATATCAGTCCTAGTACATCA  CCGCTGCAGGTCGACGGATCCTTATTCCCGGGGACGAGC  ATGGCCATGGAGGCCGAATTCATGGGATACTCCAATATTGCTTTAATC  CCGCTGCAGGTCGACGGATCCTTAGACATCAGTTGGAAGTTCCAACT  ATGGCCATGGAGGCCGAATTCATGGCATCATCAATTGTACAAAATG  CCGCTGCAGGTCGACGGATCCCTAGCAGATTGGAAGAGGAGCAC  ATGGCCATGGAGGCCGAATTCATGGCTGTAGGTGTAAGGCACTG  CCGCTGCAGGTCGACGGATCCCTAAATACGATGCGGCAGGC  ATGGCCATGGAGGCCGAATTCATGACTGAATACAGCTTGCCCAC  CCGCTGCAGGTCGACGGATCCTTAGTGTGTTTCAGCAATTTTCGAT  ATGGCCATGGAGGCCGAATTCATGGCACTTGCTAAAGAAATTATGG  CCGCTGCAGGTCGACGGATCCTCATATCGATACACTATTTGGAACACC  ATGGCCATGGAGGCCGAATTCATGGCATCAACTTCTCTTTCTCTTC  CCGCTGCAGGTCGACGGATCCTCAAAAACTGGCTCTTCTCAGAGA  ATGGCCATGGAGGCCGAATTCATGGCCACTGTTTCCTCAGCC  CCGCTGCAGGTCGACGGATCCTTAATTAGTGTAATTTTTCAGTGCGG  ATGGCCATGGAGGCCGAATTCATGGAGGAACGGAACTCAACG  CCGCTGCAGGTCGACGGATCCCTATTCAGCGAGAAGGTAAGTTGGG  ATGGCCATGGAGGCCGAATTCATGGGGTCATCGGAAAATATGG  CCGCTGCAGGTCGACGGATCCCTAGAAATATTGCTCAGTTTTAACAAATTG  GTGGGCAAGCTGTATTCAGTCATGAATTCGGCCTCCATGGCCAT  CCGCTGCAGGTCGACGGATCCGTGTGTTTCAGCAATTTTCGATG  AATTACTATTTACAATTACGGATCATGGAGAATTATGCAACAATATTTCCA  CTGTTCACCGTTAATCAAACCCATAAAGGAATTATAGATTTGCATTTGAC  TGGTGTTACTAGAGAAATAA  TCCATAAACACAGCAAAAAAAC  GAAAAGCTTGAATTCGAGCTCTGGTGTTACTAGAGAAATAA  AGCACATGCCTCGAGGTCGACTCCATAAACACAGCAAAAAAAC  GAAAAGCTTGAATTCGAGCTCTGGTGTTACTAGAGAAATAATTCTATTAAAAGC  AGCACATGCCTCGAGGTCGACTCTTTTAGAAAAAAGTTTTGCATTGAC  GAAAAGCTTGAATTCGAGCTCGTTTGTATAATACCATTACCACCTAACTGA  AGCACATGCCTCGAGGTCGACAGATTTTTTACTTTATTGTATAAGCTTTTAGTCA  GAAAAGCTTGAATTCGAGCTCCTTTAGCGGGAGCATATTTTATTTC  AGCACATGCCTCGAGGTCGACCCCACTAGAAAGGAAAGGAATTGT  GAAAAGCTTGAATTCGAGCTCCAAAAAATTCAAGAAAACGACTAAAGT  AGCACATGCCTCGAGGTCGACAACACAGCAAAAAAACAGGTGAAA  CGCTCTAGAACTAGTGGATCCATGGAGAATTATGCAACAATATTTCC  GATAAGCTTGATATCGAATTCAAAGGAATTATAGATTTGCATTTGACTC  CTATAGGGCGAATTGGGTACCTGGTGTTACTAGAGAAATAATTCTATTAAAAGC  TGTTTTTGGCGTCTTCCATGGTCCATAAACACAGCAAAAAAACAGG |
