# Supplementary material for: A WRKY transcription factor, SlWRKY75, positively regulates tomato (Solanum lycopersicum L.) resistance to Ralstonia solanacearum
Source: Front Plant Sci. 2025 Oct 30;16:1704937. doi: 10.3389/fpls.2025.1704937 (PMC12611961; doi:10.3389/fpls.2025.1704937)
Supplement: Supplementary file 4 [file Table2.doc]

Table S2. Primers used for quantitative real-time PCR (qRT-PCR)

| Primer | Sequence (5′－3′) |
| --- | --- |
| SlACTIN-F  SlACTIN-R  qSlWRKY75-F  qSlWRKY75-R  qSlNPR1-F  qSlNPR1-R  qSlTGA-F  qSlTGA-R  qSlPR1-F  qSlPR1-R  qSlPAL-F  qSlPAL-R  qSlICS-F  qSlICS-R  qSlMYC2-F  qSlMYC2-R  qLOXD-F  qLOXD-R  qSlAOS-F  qSlAOS-R  qSlAOC-F  qSlAOC-R  qSlCOI1-F  qSlCOI1-R  qSlJAZ-F  qSlJAZ-R | CTCTACATACTTGAGAGGTGCC  AGACGAGGAGAAAACATCACAA  GTAGTTACTACTTATGAAGGCATGC  GCATTTGACTCAAAATGTGCTC  ACAAGTTGATGGCACGTCTG  CCGATTCAAGTGCTCCTCTT  TACGGCAGGCTGATAACCTT  AGCTCAGTGCTCGAAGCCTA  TACTCAGGTGGTGTGGCGTA  ATGGACGTTGTCCTCTCCAG  TCAAGGCAGCTCAGAAGCTC  CTTAGTTGCTGCACGGATGA  ATGTATGCTGGTCCTGTTGG  ACCAAAGCCGACCTTATTCC  TCGGTGTCATCACCTGCTTA  CTTCTTCATCAACCGCATCA  GGCTTGCTTTACTCCTGGTC  AAATCAAAGCGCCAGTTCTT  CATCATCATCGTCATCAC  GAAGTAATCAAGTCTGTCTT  CTATCTTCTGCCTTCCAA  TGTTAGTTGAATCTGTTGAG  GAGGAACGGAACTCAACGAG  ATACCAACGATACCGCGTCT  ATCAACCAGAGAAGGCAC  TACCAAAACTCACACCAG |
